# Recommendations for a Core Outcome Set for Measuring Standing Balance in Adult Populations: A Consensus-Based Approach

**DOI:** 10.1371/journal.pone.0120568

**Published:** 2015-03-13

**Authors:** Kathryn M. Sibley, Tracey Howe, Sarah E. Lamb, Stephen R. Lord, Brian E. Maki, Debra J. Rose, Vicky Scott, Liza Stathokostas, Sharon E. Straus, Susan B. Jaglal

**Affiliations:** 1 Department of Community Health Sciences, University of Manitoba, Winnipeg, Canada; 2 Toronto Rehabilitation Institute- University Health Network, Toronto, Canada; 3 School of Health and Life Sciences, Glasgow Caledonian University, Glasgow, United Kingdom; 4 Centre for Rehabilitation Research, Nuffield Department of Orthopaedics, Rheumatology and Musculoskeletal Sciences, University of Oxford, Oxford, United Kingdom; 5 Neuroscience Research Australia, University of New South Wales, Sydney, Australia; 6 University of Toronto, Toronto, Canada; 7 California State University Fullerton, Fullerton, California, United States of America; 8 Faculty of Medicine, School of Population and Public Health, University of British Columbia, Vancouver, Canada; 9 British Columbia Injury Research and Prevention Unit and Ministry of Health, Victoria, Canada; 10 Canadian Centre for Activity and Aging, Western University, London, Canada; Cardiff University, UNITED KINGDOM

## Abstract

**Background:**

Standing balance is imperative for mobility and avoiding falls. Use of an excessive number of standing balance measures has limited the synthesis of balance intervention data and hampered consistent clinical practice.

**Objective:**

To develop recommendations for a core outcome set (COS) of standing balance measures for research and practice among adults.

**Methodology:**

A combination of scoping reviews, literature appraisal, anonymous voting and face-to-face meetings with fourteen invited experts from a range of disciplines with international recognition in balance measurement and falls prevention. Consensus was sought over three rounds using pre-established criteria.

**Data sources:**

The scoping review identified 56 existing standing balance measures validated in adult populations with evidence of use in the past five years, and these were considered for inclusion in the COS.

**Results:**

Fifteen measures were excluded after the first round of scoring and a further 36 after round two. Five measures were considered in round three. Two measures reached consensus for recommendation, and the expert panel recommended that at a minimum, either the Berg Balance Scale or Mini Balance Evaluation Systems Test be used when measuring standing balance in adult populations.

**Limitations:**

Inclusion of two measures in the COS may increase the feasibility of potential uptake, but poses challenges for data synthesis. Adoption of the standing balance COS does not constitute a comprehensive balance assessment for any population, and users should include additional validated measures as appropriate.

**Conclusions:**

The absence of a gold standard for measuring standing balance has contributed to the proliferation of outcome measures. These recommendations represent an important first step towards greater standardization in the assessment and measurement of this critical skill and will inform clinical research and practice internationally.

## Introduction

Standing balance, defined as the ability to keep the center of mass within the base of support [1], is a prerequisite for many functional activities such as mobility and fall avoidance [2, 3]. Balance impairment is common across multiple populations and leads to the greatest losses in years of healthy life and quality of life in people living with stroke [4], brain injury [5], arthritis [6], and up to 75% of people of advancing age (≥70 years) [7]. Exercise is postulated to improve balance and is associated with increased mobility and reduced falls in many of these populations [8–11]. However, synthesizing evidence on the effects of interventions for improving balance has been hampered by the extensive variation in the use of balance outcome measures among studies [2, 12]. For example, a systematic review on the effectiveness of exercise interventions to improve balance in older adults identified 95 eligible studies [2] but was able to pool less than 50% of included studies because over 25 different measures were used to assess balance. Varied use of balance measures is also seen in clinical practice, as illustrated in one survey of balance assessment practices among Canadian physiotherapists that reported use of over 20 different measures [13].

Such inconsistency in use of balance measures reflects the absence of a gold standard method for evaluating standing balance [14] and subsequent prolific development of measures [15]. This plethora highlights the complex multifactorial nature of balance; measures vary in purpose, specific components of balance evaluated, measurement techniques, target population and extent of psychometric evaluation. However, given the importance of standing balance in fall prevention and mobility enhancement, there is a need for greater consistency in standing balance measurement across studies and for individual assessments [16]. One approach to achieve a more standardized practice is to identify and recommend a core outcome set for measuring standing balance. A core outcome set (COS) is defined as a recommended minimum set of outcomes or outcome measures for a particular health construct, condition, or population, the results of which should be reported for all trials pertaining to that issue [17]. In all cases, COS recommendations do not imply that measurement of the construct should be restricted to the COS; rather, the purpose is to advocate that the COS forms a consistent component of measurement and it is expected that additional measures may also be used.

The objective of this project was to propose recommendations for a COS of standing balance measures for research and practice settings in adult populations. Although core outcome sets were originally developed for clinical trials, including health care practice in the scope of a COS offers the opportunity to expand the utility of recommendations and potential for broad uptake. Recommendation of a few representative and feasible measures that can be widely used across a range of populations and settings can facilitate evaluating the efficacy of interventions to improve standing balance, and thus a recommended COS for standing balance will directly and substantially inform clinical research and practice internationally. In turn, this will optimize the development and implementation of evidence-based exercise programs for mobility enhancement and fall prevention worldwide.

## Methods

### Design

We used a consensus-based approach incorporating a modified Nominal Group Technique based on the RAND/UCLA Appropriateness Method [18], involving a combination of anonymous rating and face-to-face group discussion [19]. These techniques have been used to develop COSs for other health outcome measures [20, 21], and published guidelines for reporting the development of COSs [17] were followed. The project was funded by a Canadian Institutes of Health Research (CIHR) planning grant (# MAG133935), and was registered on the COMET (Core Outcome Measures in Effectiveness Trials) Initiative database (available at http://www.comet-initiative.org/studies/details/244?result=true). Given the secondary nature of the data extraction, analysis, and recommendations, and as is common practice in COS development work, research ethics approval was not sought.

### Expert panel sampling and recruitment

A purposive and iterative approach was used to identify individuals to sit on an international panel of experts for the consensus process. “Experts” were operationally defined as individuals who have national or international recognition in the fields of balance, mobility, exercise or fall prevention, and who regularly evaluate balance in their work. Within this context, individuals were strategically identified to represent a range of 1) related expertise (postural control, fall prevention, geriatrics, neurology, orthopedics, health service delivery, knowledge translation); 2) professional backgrounds (bioengineering, epidemiology, kinesiology, medicine, nursing, physiotherapy); and 3) practice settings (primary care, rehabilitation, nursing homes, homecare, community). The four members of the research team who initiated the project (KMS, SBJ, BEM, SES) have established track records in postural control, fall prevention, geriatrics, and hip fracture. They worked together to identify potential panel members who collectively represented all of the target expertise, professional backgrounds and practice settings identified as relevant to balance measurement. An initial cohort of individuals identified by the research team were contacted through email by the principal investigator (KMS), informed about the project, and invited to participate. Those who declined where asked to recommend other appropriate individuals, and any suggestions were discussed by the research team prior to invitation. Individuals were not excluded if they were the developer of one of the measures under consideration, but all panel members declared at the meeting whether they had any conflicts of interest related to participating in balance COS recommendations (including authorship) of measures under consideration for the balance COS. A panel size between twelve and eighteen individuals was sought, which falls within recommended ranges for consensus panels to provide good validity without excessively affecting group processes [22]. Consent was implied when individuals agreed to join the expert panel.

### Identification of measures for consideration

A scoping review identifying published standing balance measures for adult populations [23] formed the pool of measures to be considered for the COS recommendations. Full details of the review are available. In brief, electronic searches of Medline, Embase, and CINAHL databases up to March 2014 were conducted using key word combinations of postural balance/ equilibrium, psychometrics/ reproducibility of results/ predictive value of tests/ validation studies, instrument construction/ instrument validation, geriatric assessment/ disability evaluation, as well as grey literature [24] and hand searches. Inclusion criteria were measures with a stated objective to assess balance, adult populations (aged 18 years and over), at least one psychometric evaluation, one standing balance task, a standardized protocol and evaluation criteria, and published in English. Two research assistants independently identified studies for inclusion and extracted characteristics (levels of measurement, scoring properties etc.), and psychometric properties for each measure. Two reviewers independently coded components of balance evaluated in each measure using the Systems Framework for Postural Control [25], a widely recognized model of balance. To avoid considering obsolete measures, electronic searches of Pubmed and Google Scholar were conducted on all identified measures published prior to 2009, and those with no references in peer-reviewed publications since 2009 or reported in a 2011 Canadian survey of balance assessment practices [13] were excluded.

### Consensus process

**Fig 1:**
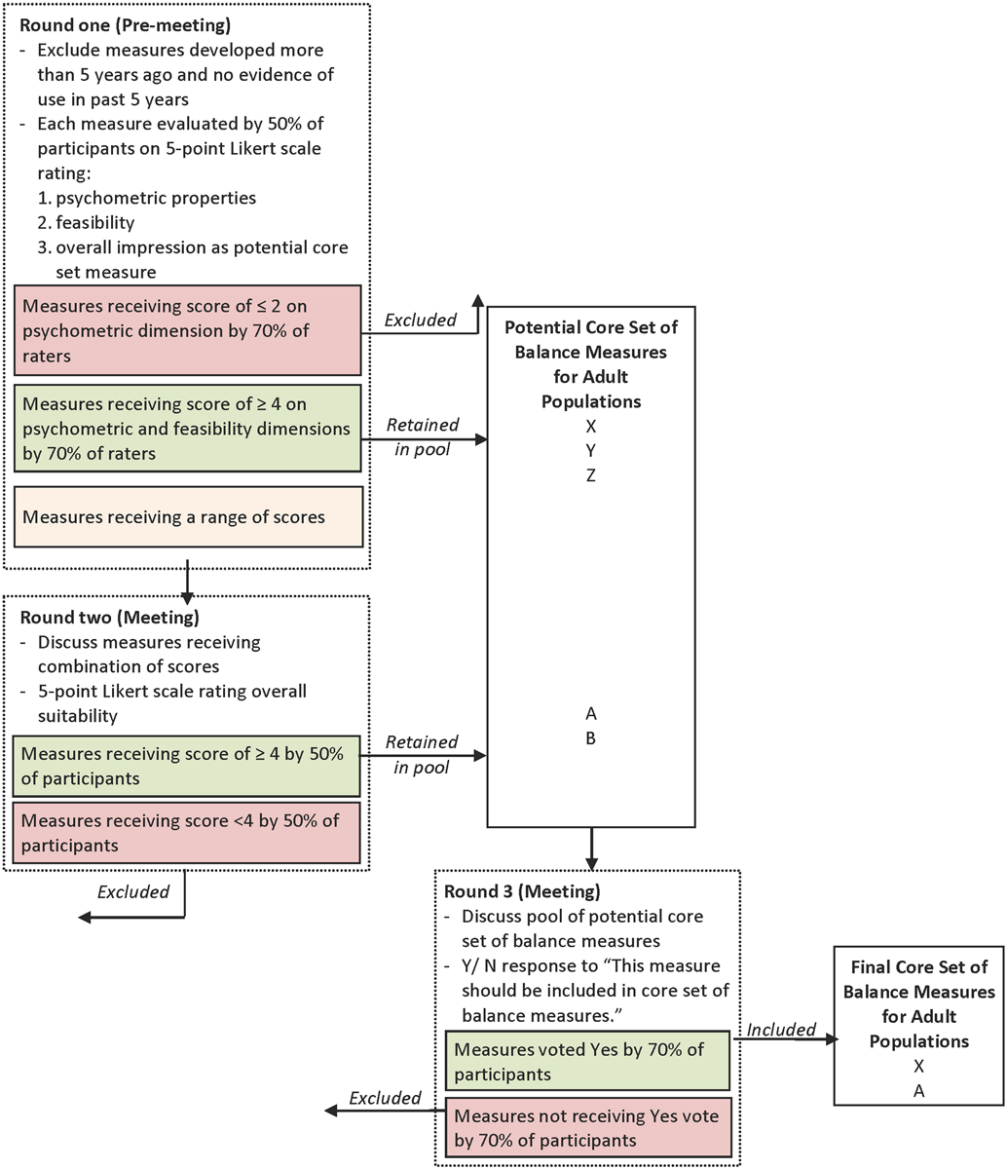
Overview of consensus process

The consensus process is summarized in Fig. 1.

#### Round One

Round one scoring took place online. To inform their scores, members of the expert panel were provided with background information, including: (i) the original publication and test items for each measure; (ii) a description of the established psychometric properties for each measure, downloaded from the Rehabilitation Measures Database, a searchable website containing evidence-based summaries of more than 250 rehabilitation measures (www.rehabmeasures.org), or a psychometric summary prepared by KMS if one was not available; (iii) results of the scoping review findings including measure characteristics and components of balance evaluated in each measure [23]; and (iv) a publication of balance assessment practices among Canadian physiotherapists [13].

Each measure was scored on a 5-point Likert scale (1- lowest, 5- highest) on three dimensions: (i) *psychometric properties (*validity, reliability etc.); (ii) *feasibility* of use on a large scale (practicality of administration, time, cost, equipment needs); and (iii) overall impression as a potential balance COS measure for adult populations. To manage workload, each measure was scored by half of the panel members, and to reduce bias, each participant had a different, randomly assigned set of measures to score. Panel members were invited to propose additional measures they felt warranted consideration.

Measures that received scores ≥ 4/ 5 on both psychometric and feasibility dimensions by 70% of scorers in round one were retained in the pool of potential COS measures and forwarded for discussion in round three. Measures that received scores ≤ 2/ 5 on the psychometric properties dimension by 70% of scorers were excluded. The remaining measures that received a range of scores across both dimensions were held for discussion in round two.

#### Round Two

Subsequent rounds took place at face-to-face meetings held in Toronto, Canada on May 29^th^ and 30^th^, 2014. One week prior to the meeting, panel members received a report of the round one results, including detailed reports of the scoring distribution and comments for each measure (S1 File). The proceedings were led by a professional facilitator with a background in physiotherapy, and were audio recorded and transcribed verbatim along with detailed notes taken by a recorder. One panel member (TH) published meeting status updates throughout the proceedings via Twitter, which are archived and available online (https://storify.com/MSK_Elf/recommending-a-core-outcome-set-for-standing-balan). In round two, measures that received a range of scores across both dimensions were discussed by the expert panel, and then each member scored each of those measures on a single 5-point Likert scale rating the *overall suitability* for inclusion in the balance COS. A discussion of the constructs important for overall suitability of a balance COS was undertaken using the OMERACT filter (Outcome Measures in Rheumatology) filter framework to guide the discussion (Table 1). The OMERACT filter is a framework of constructs developed for rheumatology core outcome sets that emphasizes the concepts of “truth”, “discrimination”, and “feasibility” [26]. Following the discussion, panel member scored each measure electronically using a web-based tool, and were blind to each other’s scores. At this phase, measures receiving scores ≥ 4/5 on overall suitability by 70% of panel members were retained in the pool of potential balance COS measures and discussed in round three.

**Table 1:** OMERACT (Outcome Measures in Rheumatology) filter to determine applicability of a measurement instrument in a setting.

Construct	Explanation
Truth	Is the measure truthful, does it measure what is intended? Is the result unbiased and relevant? The word “truth” captures issues for face, content, and construct validity (As gold standards are often not available, criterion validity is mostly not tested)
Discrimination	Does the measure discriminate between situations of interest? The situations can be states at one time (for classification or prognosis) or states at different times (to measure change). The word “discrimination” captures issues of reliability and sensitivity to change
Feasibility	Can the measure be applied easily, given constraints of time, money, and interpretability? The word “feasibility” captures an essential element in the selection of measure, one that may be decisive in determining a measure’s success

#### Round Three

In round three, panel members discussed the measures forwarded from rounds one and two. They also discussed and agreed that any panel members who developed measures under consideration in round three would abstain from the discussion and final vote. In round three, panel members responded to the following yes/ no statement for each measure: “This measure should be included in a COS of balance measures for adult populations”. Measures required support by a minimum of 70% of the panel members to be included in the final balance COS recommendations.

## Results

### Expert panel membership

Twenty individuals were invited to join the expert panel in the consensus exercise. Two declined the invitation, and four who accepted the invitation withdrew prior to the beginning of consensus activities due to scheduling conflicts. Fourteen individuals (70% of those invited) joined the expert panel—13 in person and one via teleconference (KV). One co-investigator participated in discussions via teleconference but did not vote (BEM). Expert panel characteristics are described in Table 2. Four panel members declared that they developed measures under consideration for the balance COS (KB, FH, EI, DR).

**Table 2:** Expert panel participant characteristics.

Participant	Country	Primary Appointment	Affiliation Setting	Academic Background	Subject area expertise	Population expertise	Setting expertise	Years of experience
*K. Berg	Canada	Chair, Department of Physical Therapy	University	Epidemiology, Physical Therapy	Fall prevention, health service delivery	Geriatrics	Acute care, nursing home, home care	40
*F. Horak	USA	Professor of Neurology	University	Physical Therapy, Neurophysiology	Postural control, outcome measures	Neurology	Rehabilitation	40
**T. Howe**	UK	Professor of Rehabilitation Sciences	University	Physical Therapy	Exercise, postural control, outcome measures	Geriatrics, neurology, musculoskeletal	Rehabilitation	30
**E. Inness**	Canada	Clinic Leader—Balance, Mobility & Falls Clinic	Rehabilitation Hospital	Physical Therapy	Falls prevention, postural control, outcome measures	Neurology	Rehabilitation	25
**S. Jaglal**	Canada	Professor and Chair in Rehabilitation Research	University	Epidemiology	Health service delivery, hip fracture, knowledge translation	Musculoskeletal	Rehabilitation	20
**S. Lamb**	UK	Professor of Rehabilitation Sciences	University	Physical Therapy	Falls prevention, health service delivery, knowledge translation, postural control	Geriatrics, musculoskeletal	Acute care, rehabilitation, primary care, community	25
**S. Lord**	Australia	Senior Principal Research Fellow	Medical Research Institute	Epidemiology, Physiology, Psychology	Falls prevention, knowledge translation, postural control	Geriatrics, neurology, musculoskeletal	Acute care, rehabilitation, community	30
**S. MacKay**	Canada	Manager, Research & Evaluation	Home Care	Kinesiology	Falls prevention, health service delivery	Geriatrics	Home care	8
**B. Maki	Canada	Senior Scientist	Rehabilitation Hospital	Kinesiology, Biomechanics, Biomedical Engineering	Falls prevention, postural control,	Geriatrics	Rehabilitation, community	30
**D. Rose**	USA	Director, Institute of Gerontology and Center for Successful Aging	University	Kinesiology	Fall prevention, knowledge translation, postural control	Geriatrics	Rehabilitation, community, home care	29
**V. Scott**	Canada	Senior Advisor, Fall and Injury Prevention, BC Injury Research & Prevention Unit and Ministry of Health	Research Institute	Nursing, Health Policy	Fall prevention, health service delivery, knowledge translation	Geriatrics	Primary care, nursing homes, community, home care	20
**K. Sibley**	Canada	Postdoctoral Fellow	Research Institute	Kinesiology	Fall prevention, knowledge translation, postural control	Geriatrics, neurology, musculoskeletal	Rehabilitation	10
**D. Skelton**	UK	Professor of Ageing and Health	University	Exercise Physiology	Fall prevention, health services delivery, postural control, exercise interventions	Geriatrics	Primary care, nursing homes, community	24
**L. Stathokostas**	Canada	Researcher	University	Kinesiology	Aging, exercise	Geriatrics	community	15
**K. Van Ooteghem**	Canada	Postdoctoral Fellow	University	Kinesiology	Postural control	Geriatrics	Nursing home, community	7

* Did not participate in round three discussion and vote.

** Did not vote.

### COS development

**Fig 2:**
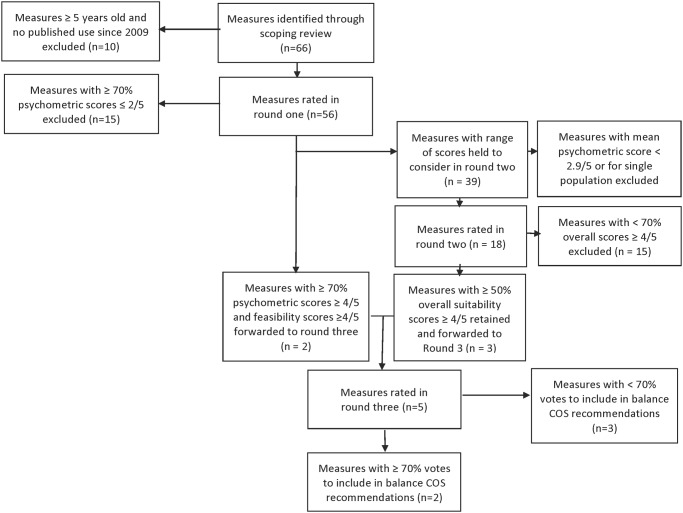
Overview of standing balance COS development results.

The results are summarized in Fig. 2. The scoping review identified 66 measures. Of these, ten measures published at least five years earlier with no evidence of use since then were excluded. Fifty-six measures were considered in the pool of potential balance COS measures (Table 3) and scored in round one. Following round one, 15 measures were excluded, two measures were forwarded to round three (Berg Balance Scale (BBS) [27] and Timed Up-and-Go (TUG) Test [28]), and 39 measures were held for discussion in round two.

**Table 3:** Measures considered for standing balance COS (n = 56).

Measure	Result
Activity-based Balance Level Evaluation (ABLE) Scale [Ardolino et al. Phys Ther. 2012]	Excluded in round one (consensus on low psychometric score)
Balance Computerized Adaptive Testing (CAT) System [Hsueh et al. Phys Ther. 2010]	Excluded in round two (not discussed, specific to stroke)
Hierarchical Balance Short Forms (HBSF) [Hou et al. Arch Phys Med Rehabil. 2011]	Excluded in round one (consensus on low psychometric score)
Balance Error Scoring System (BESS) [Riemann et al. J Sport Rehabil. 1999]	Excluded in round two (insufficient consensus on overall suitability)
Modified Balance Error Scoring System (M-BESS) [Hunt et al. Clin Journal Sport Med. 2009]	Excluded in round one (consensus on low psychometric score)
Balance Evaluation Systems Test (BESTest) [Horak et al. Phys Ther. 2009]	Excluded in round two (insufficient consensus on overall suitability)
Brief Balance Evaluation Systems Test (Brief BESTest) [Padgett et al. Phys Ther 2012]	Excluded in round two (insufficient consensus on overall suitability)
Mini Balance Evaluation Systems Test (Mini BESTest) [Franchignoni et al. J Rehabil Med 2010]	Included in balance COS recommendations
Balance Outcome Measure for Elder Rehabilitation (BOOMER) [Haines et al. Arch Phys Med Rehabil. 2007]	Excluded in round two (not discussed, mean psychometric score < 2.9)
BDL Balance Scale [Lindmark et al. Advances in Physiotherapy. 2012]	Excluded in round two (not discussed, mean psychometric score < 2.9)
Berg Balance Scale (BBS) [Berg et al. Physiotherapy Canada. 1989]	Included in balance COS recommendations
Short Form of the Berg Balance Scale (SFBBS) [Chou et al. Phys Ther. 2006]	Excluded in round two (insufficient consensus on overall suitability)
Short Berg Balance Scale [Hohtari-Kivimaki et al. Aging-Clinical & Experimental Research. 2012]	Excluded in round two (insufficient consensus on overall suitability)
Brunel Balance Assessment (BBA) [Tyson et al. Clin Rehabil. 2004]	Excluded in round two (not discussed, mean psychometric score < 2.9)
Clinical Gait and Balance Scale (GABS) [Thomas et al. J Neurol Sci. 2004]	Excluded in round one (consensus on low psychometric score)
Clinical Test of Sensory Interaction in Balance (CTSIB)	Excluded in round two (not discussed, mean psychometric score < 2.9)
Community Balance and Mobility Scale (CB&M) [Howe et al. Clin Rehabil. 2006]	Excluded in round two (insufficient consensus on overall suitability)
Dynamic Balance Assessment (DBA) [Desai et al Phys Ther. 2010]	Excluded in round two (not discussed, mean psychometric score < 2.9)
Dynamic Gait Index [Shumway-Cook et al. Phys Ther. 1997]	Excluded in round two (insufficient consensus on overall suitability)
Four-item Dynamic Gait Index (4-DGI) [Marchetti et al. Phys Ther. 2006]	Excluded in round two (not discussed, mean psychometric score < 2.9)
Functional Gait Assessment (FGA) [Wrisley et al. Phys Ther. 2004]	Excluded in round two (insufficient consensus on overall suitability)
Five Times Sit-to-Stand Test (5-STS) [Whitney et al. Phys Ther. 2005]	Excluded in round two (insufficient consensus on overall suitability)
Four Square Step Test (FSST) [Dite and Temple. Arch Phys Med Rehabil. 2002]	Excluded in round two (not discussed, mean psychometric score < 2.9)
Fullerton Advanced Balance (FAB) Scale [Rose et al. Arch Phys Med Rehabil. 2006]	Excluded in round two (insufficient consensus on overall suitability)
Functional Reach Test [Duncan et al. J Gerontol. 1990]	Excluded in round two (insufficient consensus on overall suitability)
Multidirectional Reach Test [Newton. J Gerontol A Biol Sci Med Sci. 2001]	Excluded in round two (insufficient consensus on overall suitability)
Hierarchical Assessment of Balance and Mobility (HABAM) [MacKnight and Rockwood. Age & Ageing 1995]	Excluded in round two (insufficient consensus on overall suitability)
Limits of Stability Test (LOS) [Clark et al. Arch Phys Med Rehabil. 1997]	Excluded in round two (not discussed, mean psychometric score < 2.9)
Modified Figure of Eight Test [Jarnlo and Nordell. Phys Theor Pract. 2003]	Excluded in round one (consensus on low psychometric score)
Parallel Walk Test (PWT) [Johansson et al. Phys Theor Pract. 1991]	Excluded in round one (consensus on low psychometric score)
Performance Oriented Mobility Assessment (POMA) [Tinetti. J Am Geriatr Soc. 1986]	Excluded in round two (insufficient consensus on overall suitability)
Modified Performance Oriented Mobility Assessment [Fox et al. Arch Phys Med Rehabil. 1996]	Excluded in round one (consensus on low psychometric score)
Postural Assessment Scale for Stroke Patients (PASS) [Benain et al. Stroke. 1999]	Excluded in round one (consensus on low psychometric score)
Short Form of Postural Assessment Scale for Stroke Patients (SFPASS) [Chien et al. Neurorehabil Neur Repair. 2007]	Excluded in round two (not discussed, specific to stroke)
Pull/ Retropulsion Test [Visser et al. Arch Phys Med Rehabil. 2003]	Excluded in round two (not discussed, mean psychometric score < 2.9)
Push and Release Test [Jacobs et al. J Neurol. 2006]	Excluded in round two (not discussed, mean psychometric score < 2.9)
Rapid Step Test (RST) [Medell et al. J Geron A Biol Sci Med Sci. 2000]	Excluded in round one (consensus on low psychometric score)
Sensory Organization Test (SOT) [Ford-Smith et al. Arch Phys Med Rehabil. 1995]	Excluded in round two (not discussed, mean psychometric score < 2.9)
Head-Shake Sensory Organization Test (HS-SOT) [Pang et al. Phys Ther. 2011]	Excluded in round one (consensus on low psychometric score)
Short Physical Performance Battery (SPPB) [Guralnik et al. J Gerontol. 1994]	Excluded in round three (insufficient consensus)
Side-Step Test [Fujisawa et al. Clin Rehabil. 2006]	Excluded in round two (not discussed, mean psychometric score < 2.9)
Single Leg Hop Stabilization Test [Riemann et al. J Sport Rehabil. 1999]	Excluded in round one (consensus on low psychometric score)
Single leg Stance Test [Bohannon. Topics Geri Rehabil. 2006]	Excluded in round two (not discussed, mean psychometric score < 2.9
Spring Scale Test (SST) [DePasquale and Toscano. J Geri Phys Ther. 2009]	Excluded in round two (not discussed, mean psychometric score < 2.9)
Standing Test for Imbalance and Disequilibrium (SIDE) [Teranishi et al. Jap J Comp Rehabil Sci. 2010]	Excluded in round one (consensus on low psychometric score)
Star Excursion Balance Test (SEBT) [Hertel et al. J Sport Rehabil. 2000]	Excluded in round one (consensus on low psychometric score)
Step Test (ST) [Hill et al. Physiotherapy Canada. 1996]	Excluded in round two (not discussed, mean psychometric score < 2.9)
Tandem Stance [Hile et al. Phys Ther 2012]	Excluded in round two (not discussed, mean psychometric score < 2.9)
Timed Up-and-Go Test (TUG) [Podsiadlo et al. J Am Geriatr Soc. 1991]	Excluded in round three (forwarded directly from round one)
Expanded Timed Up-and-Go Test (ETUG) [Botolfsen et al. Phys Res Int. 2008]	Excluded in round two (insufficient consensus on overall suitability)
TURN180 [Simpson et al. Physiotherapy. 2002]	Excluded in round two (not discussed, mean psychometric score < 2.9)
Unified Balance Scale [La Porta et al. J Rehabil Med. 2011]	Excluded in round three (insufficient consensus)
Timed Up-and-Go Assessment of Biomechanical Strategies (TUG-ABS) [Faria et al. J Rehabil Med. 2013	Excluded in round one (consensus on low psychometric score)
Posture and Posture Ability Scale (PPAS) [Rodby-Bousquet et al. Clin Rehab. 2012]	Excluded in round one (consensus on low psychometric score)
High Level Mobility Assessment Tool (HiMAT) [Williams et al. Brain Inj. 2005]	Excluded in round two (insufficient consensus on overall suitability)
Cross Step Moving on Four Spots Test (CSFT) [Yamaji & Demura Arch Phys Med Rehabil 2013]	Excluded in round one (consensus on low psychometric score)

At the meeting, initial discussions focused on the parameters of the COS and feasibility of making one recommendation applicable to research and practice in all adult populations. The advantages and disadvantages of both broad and narrow-scoped recommendations were debated, and the decision was made to maintain the objective to recommend a COS for measuring standing balance in research and practice in adult populations. Subsequent discussions addressed the constructs necessary for a COS for standing balance. There was general agreement regarding the application of the OMERACT framework principles within the consideration of “overall suitability”, and the need to consider the many components that comprise the balance “system” [1].

Once these parameters were defined, the group considered the 39 measures held for discussion in round two. While these measures did not meet either of the *a priori* criteria for exclusion or forwarding, in order to expedite the consensus process the expert panel agreed to exclude 21 measures with either a mean psychometric properties score < 2.9 or validated in only one adult population. As a result of this discussion, 21 measures were excluded and 18 were individually discussed and scored (S2 File). Following the round two vote, only one measure (Mini Balance Evaluation Systems Test [Mini BESTest] [29]) reached the 70% threshold for forwarding to round three. However, to promote discussion the group agreed to forward two additional measures that achieved sufficient scores by at least 50% of panel members (the Short Physical Performance Battery [30] and Unified Balance Scale [31]). As such, three measures were forwarded to round three and fifteen measures were excluded.

Five measures were discussed in round three: two that were forwarded directly from round one, and three that were forwarded from round two. The five measures considered in round three were: BBS, Mini BESTest, Short Physical Performance Battery, TUG Test, and Unified Balance Scale. Two panel members were developers of two of the measures under consideration, and abstained from the discussion and vote. As such, twelve panel members participated and voted in round three. In the round three discussion, comments centered on whether a single measure could achieve all the objectives of the standing balance COS in research and practice in adult populations. Comments suggested that the group thought that while a single measure would be ideal from a minimum dataset perspective, one measure could not address the full spectrum of abilities among the adult population, and that a small number of measures—less than three—would be a permissible compromise. Of the five measures considered in round three, two achieved consensus on being included in COS recommendations for measuring standing balance in research and practice in adult populations: Berg Balance Scale [27] and Mini Balance Evaluation Systems Test [29] (S3 File).

## Discussion

The need for increased consistency and psychometric rigor in the evaluation of standing balance in adult populations in order to advance understanding and implementation of optimal interventions to improve mobility and decrease falls is well-recognized. The expert panel convened in the current project recommends that at a minimum, either the Berg Balance Scale or the Mini Balance Evaluation Systems Test should be used when measuring standing balance for research and practice in adult populations.

Both the face-to-face panel meeting and anonymous scoring were integral to the development of the recommendations. The interactive discussions allowed for debate and reflection, while anonymous voting allowed individual panel members to make a full and equal contribution to the recommendations even if they did not share the opinion of the majority. This novel project represents the first attempt to make COS recommendations for the field of balance research and practice, and as such should be both viewed as a starting point and revisited in the future.

### Two recommended measures in the standing balance COS

Two measures gained consensus for recommendation by the panel. Characteristics of both measures are presented in Table 4, and readers are encouraged to consult the Rehabilitation Measures Database for a more detailed description of the psychometric properties of each measure. Comparisons of the two measures have noted that they are highly correlated (correlation coefficients ranging from 0.79–0.94 [32–36], and in one study directly comparing the psychometric properties of the Mini BESTest and BBS, both measures performed similarly on the majority of characteristics [32].

**Table 4:** Characteristics of measures included in standing balance COS.

	Berg Balance Scale	Mini Balance Evaluation Systems Test
Year of publication	1989	2010
Number of items	14	14
Number of categories	5	3
Equipment required	Stop watch, chair with arm rests, measuring tape or ruler, object to pick up off the floor (e.g. pencil), step stool	60 cm x 60 cm block of 4" medium density Tempur foam, incline ramp of 10 degree slope, chair without arm rests or wheels, firm chair with arms, 23 cm high box, stop watch, masking tape marked on floor at 3 m from front of chair
Time to administer	15–20 minutes	10–15 minutes
Populations used with	Older adults [37, 38], multiple sclerosis [39], osteoarthritis [40], Parkinson’s Disease [41, 42], spinal cord injury [43, 44], stroke [45–48], brain injury [49], vestibular dysfunction [50]	People with neurological impairments [33, 51], people with age-related balance disorders [32], community-dwelling older adults [52]
Psychometric properties evaluated	Minimal detectable change, minimal clinically important difference, test-retest reliability, inter-rater reliability, intra-rater reliability, internal consistency, criterion validity, construct validity, responsiveness	Minimal detectable change, minimal clinically important difference, test-retest reliability, inter-rater reliability, intra-rater reliability, internal consistency, criterion validity, construct validity, responsiveness
Reported Standard Error of Measurement (SEM) range	SEM = 1.2–2.9 [32, 37, 39, 45, 53, 54]	SEM = 1.3 [32]
Reported minimal detectable change (MDC) range	MDC = 3.3–8.1 [32, 37, 39, 45, 53–56]	MDC = 3–3.5 [32, 33]
Reported minimal clinically important difference (MCID) range	n/a	MCID = 4 [32]
Reported test-retest reliability range	ICC = 0.72–0.99 [32, 39, 49, 53, 54, 56–61]	ICC = 0.80–0.96 [32, 33, 51, 60, 62]
Reported inter-rater reliability range	ICC = 0.84–0.98 [32, 44, 47, 48, 55, 57–61, 63]	ICC = 0.72–0.98 [32, 33, 51, 60, 62]
Reported internal consistency range	Chronbach’s alpha = 0.92–0.98 [32, 44, 47, 48, 56, 63–65]	Chronbach’s alpha = 0.89–0.93 [32, 33]
Reported criterion validity ranges	r = 0.90–0.95 with Fugl Meyer Assessment and Postural Assessment for Stroke Scale [47], r = 0.67–0.85 with other balance tests [32, 38, 60]	r = 0.79–0.94 with Berg Balance Scale [32–36], r = 0.55–0.83 with other balance tests [33, 34, 66]
Reported construct validity ranges	Convergent with the Barthel index r = 0.87–0.94 [47, 67]	Discriminates between stroke vs. healthy [33], faller vs. non-faller [33, 51], balance deficits vs. not [35]
Reported responsiveness ranges	Effect size = 0.26–1.11 [47, 65, 68], area under ROC curve = 0.91 [32]	Area under ROC curve = 0.92 [32]
Component of balance evaluated (23)	Underlying motor systems, static stability, dynamic stability, functional stability limits, anticipatory postural control, sensory integration	Underlying motor systems, static stability, dynamic stability, anticipatory postural control, sensory integration, reactive postural control, cognitive influences on balance, verticality

ICC = Intra-class correlation; ROC = Receiver Operating Characteristic.

The BBS was recommended because it is both well-validated in a number of adult populations and widely used in both research and practice settings. It was published in 1989, with the objective to develop a valid measure of balance that was appropriate for geriatric patients (aged 60 years and older) and for use in a clinical setting [27]. It has been widely evaluated subsequent to its initial development, and tested in a number of populations. It is commonly used in physiotherapy practice [13] and has been translated into several languages. These factors contribute to the suitability of the BBS for standing balance COS recommendations and potential for broad implementation.

A limitation of the BBS is that ceiling effects have been well-documented in higher functioning individuals [43, 56, 65, 69], restricting its suitability for all adult populations. Moreover, while it includes some components of balance, including underlying motor systems, static and dynamic stability, functional stability limits, anticipatory postural control and sensory integration, it does not evaluate verticality, reactive postural control, or cognitive influences on balance [23], which are all important for avoiding falls.

The second recommended measure in the standing balance COS, the Mini BESTest, addresses some of the limitations in the BBS. The Mini BESTest was published in 2010. It was developed as a shorter version of a more comprehensive test [70], using factor and Rasch analyses [29]. Documented ceiling effects were less than the BBS in a sample of inpatients (mean age 66 years) with balance disorders [32], however one study noted a minor ceiling effect in very high functioning neurological patients [29]. It evaluates most components of postural control: underlying motor systems; verticality; static and dynamic stability; anticipatory and reactive postural control; integration of sensory information; and cognitive influences on balance; but not functional stability limits [23].

However, as with the BBS, there are also limitations to the Mini BESTest in the context of standing balance COS recommendations. It has been evaluated considerably less than the BBS, likely related to its more recent emergence in the literature. Responsiveness has been demonstrated in prospective descriptive studies [32], but the Mini BESTest has yet to be published in a clinical trial. Moreover, there is no published evidence of its uptake in clinical or community practice. Panel members acknowledged that the Mini BESTest requires more population testing, and its applicability across care settings and functional abilities needs to be demonstrated.

These two measures received the votes required for recommendation because they collectively best represent the objectives of the standing balance COS. They have unique features that make them suitable for COS recommendations and it is recommended that users choose at least one of these measures based on their particular needs. In considering which of the two measures to use in research or practice, readers may wish to consider a number of factors highlighted by the panel. The BBS may be considered more suitable for lower functioning adults, while preliminary data suggests the Mini BESTest may cover the continuum of balance abilities. If ability to perform the test is not an issue, the Mini BESTest evaluates more components of postural control than the BBS, and may be considered a more comprehensive measure.

### Measures not included in the balance COS recommendations for adult populations

The very definition of a core outcome set restricts the number of measures that can be recommended. Many well-developed balance measures were excluded from the current COS recommendations because they were too narrow in scope of target population or feasibility on a broad scale. Readers are cautioned not to infer that the current recommendations constitute best practice recommendations for balance assessment, but instead are recommended as a minimum standard for standing balance measurement. In fact, adoption of the COS measurement should not be construed as a comprehensive assessment of balance, and the panel recommends that additional population-specific measures be used, particularly when designing balance training programs.

Of the 56 measures considered for the balance COS, five reached the final round of discussion. While only two of these measures were included in the final recommendations, the three excluded measures each warrant a specific comment. The TUG test received high scores in round one and was forwarded directly to round three. In those discussions; the panel recognized its psychometric properties, feasibility and widespread clinical utility, but questioned in regards to variability in methods of application and as to whether it genuinely reflected the construct of ‘balance’. Moreover, the TUG test is also included in the Mini BESTest which was recommended in the balance COS. The Unified Balance Scale and Short Physical Performance Battery were both included in the round three discussions as a result of slightly relaxed criteria modified during the meeting, but neither achieved consensus on recommendation for the final COS. As such, the relaxed criteria did not unduly influence the outcome of the recommendations. The Unified Balance Scale, a recent scale combining items from the Balance Evaluation Systems Test [70], Fullerton Advanced Balance Scale [71], and Performance Oriented Mobility Assessment [72] also received high scores from the panel in rounds one and two, and discussions noted its comprehensive nature and appropriateness for a wide range of physical abilities [bed to community]. Its potential for future COS recommendation was noted, but the panel recognized it is not currently appropriate due to insufficient psychometric evaluation and high number of test items [27]. Finally, the Short Physical Performance Battery was discussed in round three and its psychometric properties and utility for large clinical trials was recognized. Although it did not reach consensus for inclusion in the standing balance COS recommendations, its use as a quick measure of lower extremity function that includes a standing balance item and appropriateness for large cohort and intervention studies where balance and mobility were not primary outcome measures was recognized.

### Limitations

The current standing balance COS recommendations are not without limitations, and should be interpreted in this context. First, it is acknowledged that the consensus process is not a completely objective exercise. The panel members, while invited with the goal of being representative, may not share the opinions of all potential users of the standing balance COS recommendations. While attempting to account for practice-related issues, the panel’s expertise was skewed towards research-related issues. Although attempts were made to control for conflicts of interest (such as developers of measures in contention in round three not participating in the final vote), there is no guarantee that they were eliminated. Second, the broad aims of the current standing balance COS objectives are both a strength and a weakness. There may still be some questions about applicability in some populations and/ or settings. Future iterations of balance COS recommendations may elect to narrow the scope of populations and settings included in the review, but would risk losing the ability to make meaningful comparisons across groups. Third, no single measure met all the intended objectives for the COS recommendations. As such, variation in reporting is still going to be an issue and may impact the ability to synthesize balance intervention data. The panel acknowledged this limitation, but felt the tradeoff of recommending a single balance measure was impractical and would limit successful uptake. Another consequence of the decision to recommend two measures in the standing balance COS is that the decision on what measure to use becomes more complicated and requires some discretion. Fifth, there will be a number of challenges for implementation of the recommendations. It is acknowledged that both measures require both a significant investment of time, as well as some training and equipment, which have implications for implementation. If users are not currently using one of the recommended measures, adoption of the COS recommendations will require changing their behavior, which also has implications for implementation. In particular, the Mini BESTest is less widely known, which may skew uptake towards the more familiar BBS.

## Conclusions

The lack of a gold standard measure and subsequent disparate quantity and nature of existing approaches for the measurement of standing balance are an important factor limiting the ability to advance the optimization of exercise interventions for fall prevention and mobility enhancement, and may be related to clinicians’ frustrations with outcome measures [73] and challenges prescribing exercise programs [74]. These COS recommendations for evaluating standing balance reflect an attempt to find ‘common ground’ that can meet the needs of a broad range of users. Our recommended COS for standing balance will directly and substantially inform clinical research and practice internationally. However, continued efforts to promote uptake and implementation of the COS will be required to maximize its utility.

## Supporting Information

S1 File Round One Results. This file contains the round one ranking results by measure, organized by decision. It contains basic information about each measure, the scoring distribution for the psychometric and feasibility categories, and comments noted by the panel.
(PDF)

S2 File Round Two Results. This file contains the results of the second round of voting, including the criterion calculations for moving to round three.
(PDF)

S3 File Round Three Results. This file contains the number of votes received for each measure and the decision.
(PDF)
