# Supplementary material for: Recommendations for a Core Outcome Set for Measuring Standing Balance in Adult Populations: A Consensus-Based Approach
Source: PLoS One. 2015 Mar 13;10(3):e0120568. doi: 10.1371/journal.pone.0120568 (PMC4358983; doi:10.1371/journal.pone.0120568)
Supplement: S1 File — This file contains the round one ranking results by measure, organized by decision. It contains basic information about each measure, the scoring distribution for the psychometric and feasibility categories, and comments noted by the panel. (PDF) [file pone.0120568.s001.pdf]

**Measure ID**	**2**
**Name**	**Balance Computerized Adaptive Testing (CAT) System**
**Reference**	Hsueh et al. Phys Ther. 2010
**Purpose**	To assess balance function in people with stroke
**# of Items**	34
**Evaluation Parameters**	Categorical
**# of Categories**	26 items have 2 scoring categories and 8 items have 3 scoring categories
**Graded Progression**	No

**Mean Round 1 Results**	
**Psychometric**	3.7
**Feasibility**	3.0
**Overall**	3.0

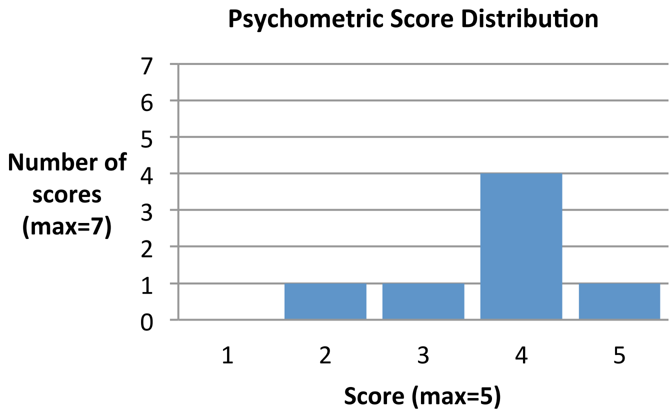

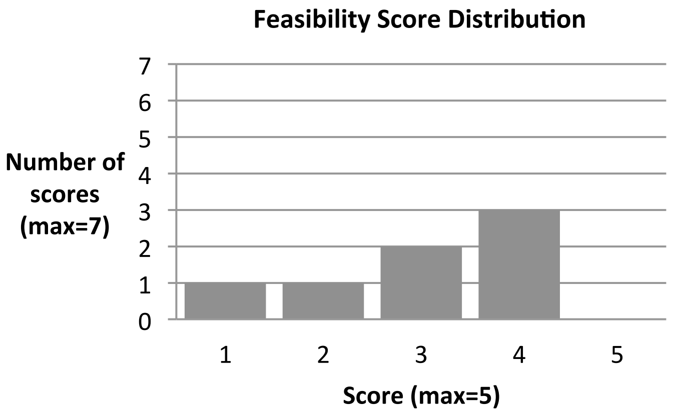

Comments from R1 Rankings: More evaluation needed • Stroke-specific so not sure of use in general population, many items refer to affected leg etc. Nothing on cost of equipment or training needed • Broad range of tests, each pass/fail. Long assessment • Need to discuss how feasible CAT system is to implement in practice •

**Measure ID**	**4**
**Name**	**Balance Error Scoring System (BESS)**
**Reference**	Riemann et al. J Sport Rehabil. 1999
**Purpose**	To assess postural stability
**# of Items**	6
**Evaluation Parameters**	Continuous (number of errors)
**# of Categories**	N/A
**Graded Progression**	No

**Mean Round 1 Results**	
**Psychometric**	3.1
**Feasibility**	3.1
**Overall**	2.5

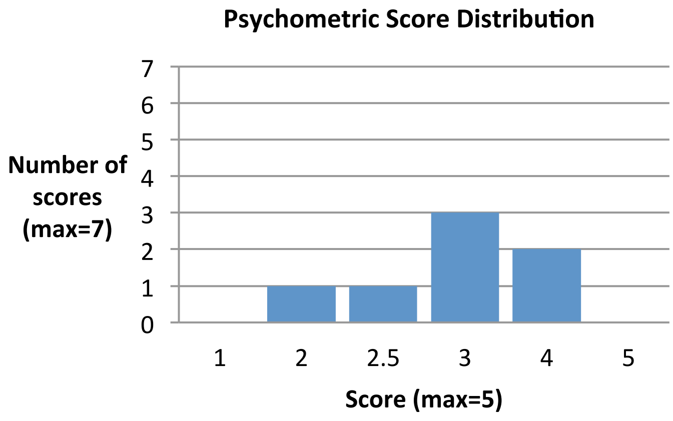

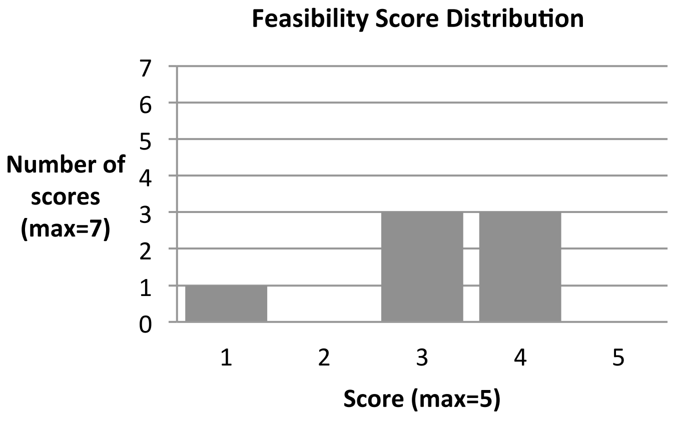

Comments from R1 Rankings: Sample was 'young varsity athletes' needs to be tested on those with balance issues • Administered to young athletes. Relies on clinician acumen in scoring • Have to buy special equipment; relevant for return to play decisions •

**Measure ID**	**6**		
**Name**	**Balance Evaluation Systems Test (BESTest)**	**Mean Round 1 Results**	
**Reference**	Horak et al. Phys Ther. 2009	**Psychometric**	3.9
**Purpose**	To help physical therapists identify underlying postural control systems that may be responsible for poor functional balance	**Feasibility**	2.3
**# of Items**	36	**Overall**	3.0
**Evaluation Parameters**	Categorical		
**# of Categories**	4		
**Graded Progression**	No		

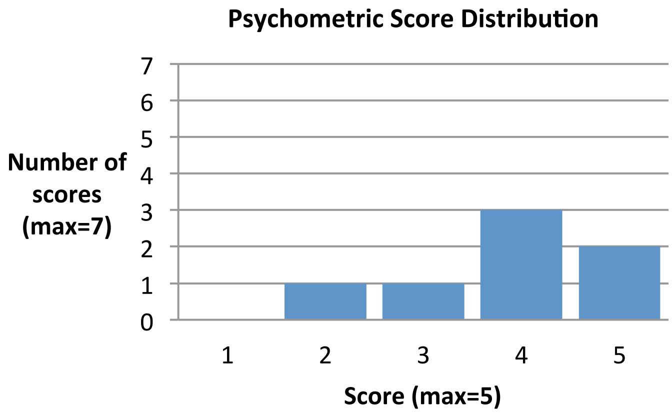

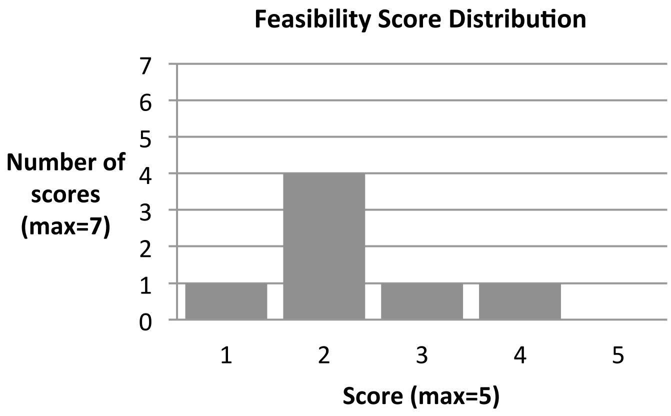

Comments from R1 Rankings: Best as follow-up tool in clinics • Long (30 min) assessment require clinician acumen and training. Broad range of complementary measures •

**Measure ID**	**7**		
**Name**	**Brief Balance Evaluation Systems Test (Brief BESTest)**	**Mean Round 1 Results**	
**Reference**	Padgett et al. Phys Ther 2012	**Psychometric**	3.3
**Purpose**	To assess balance performance in 6 specific contexts of postural control to allow for identification of specific balance systems responsible for poor balance	**Feasibility**	4.0
**# of Items**	8	**Overall**	3.7
**Evaluation Parameters**	Categorical		
**# of Categories**	4		
**Graded Progression**	No		

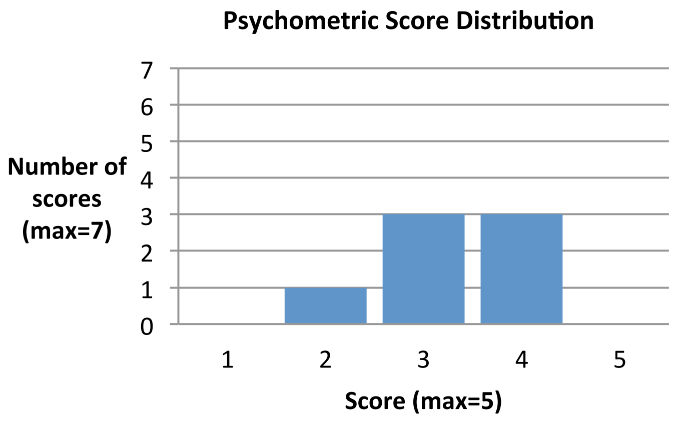

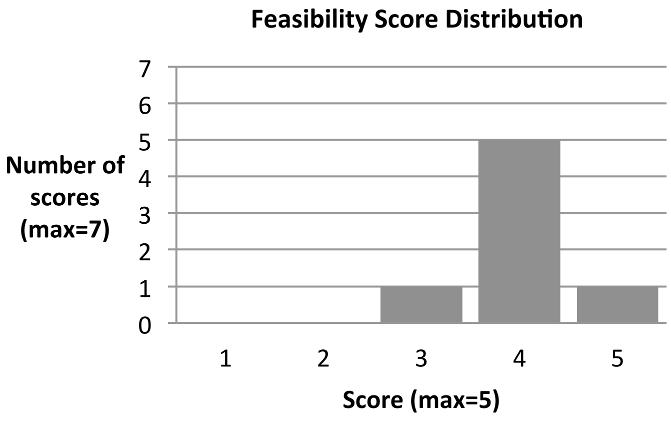

Comments from R1 Rankings: Cheap equipment, tested in 4 specific populations of LTCs, 10 mins to complete though • Short and easy to administer • Fall discrimination provided of little value •

**Measure ID**	**8**
**Name**	**Mini Balance Evaluation Systems Test (Mini BESTest)**
**Reference**	Franchignoni et al. J Rehabil Med 2010
**Purpose**	To comprehensively assess balance in a short time period
**# of Items**	14
**Evaluation Parameters**	Categorical
**# of Categories**	3
**Graded Progression**	No

Mean Round 1 Results

**Psychometric**	4.1
**Feasibility**	3.5
**Overall**	4.0

Psychometric Score Distribution

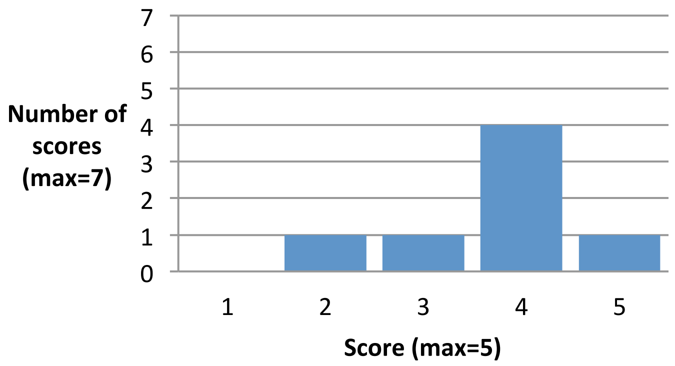

Feasibility Score Distribution

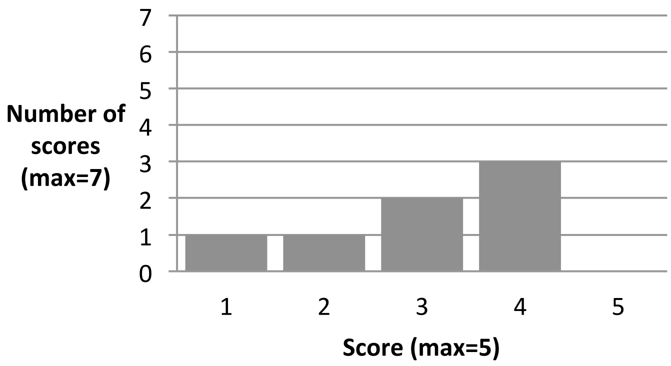

Comments from R1 Rankings: Too many items • Rasch analysis undertaken in sample with neurological conditions, not a normal sample •

**Measure ID**	**9**
**Name**	**Balance Outcome Measure for Elder Rehabilitation (BOOMER)**
**Reference**	Haines et al. Arch Phys Med Rehabil. 2007]
**Purpose**	To be a global standing balance outcome measure for elder rehabilitation
**# of Items**	4
**Evaluation Parameters**	Categorical
**# of Categories**	5
**Graded Progression**	No

Mean Round 1 Results

**Psychometric**	2.6
**Feasibility**	3.6
**Overall**	2.8

Psychometric Score Distribution

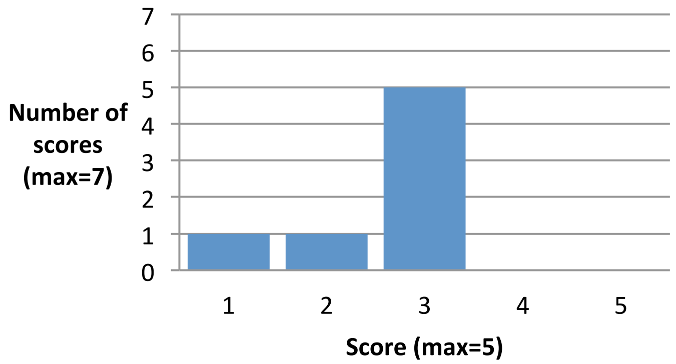

Feasibility Score Distribution

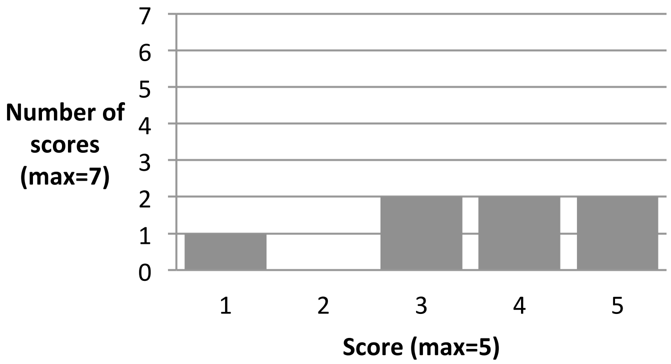

Comments from R1 Rankings: Addresses multiple dimensions • Large samples for reliability (n=784) and construct validity (n=272) •

**Measure ID**	**10**
**Name**	**BDL Balance Scale**
**Reference**	Lindmark et al. Advances in Physiotherapy. 2012
**Purpose**	To quantitatively measure balance at a relatively high level
**# of Items**	10
**Evaluation Parameters**	Categorical
**# of Categories**	5
**Graded Progression**	No

**Mean Round 1 Results**	
**Psychometric**	2.3
**Feasibility**	3.3
**Overall**	2.7

Psychometric Score Distribution

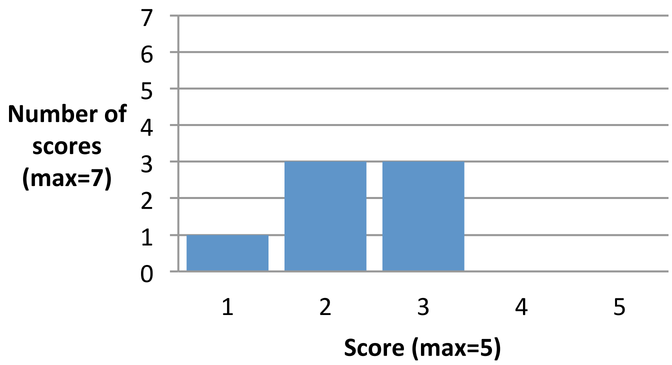

Feasibility Score Distribution

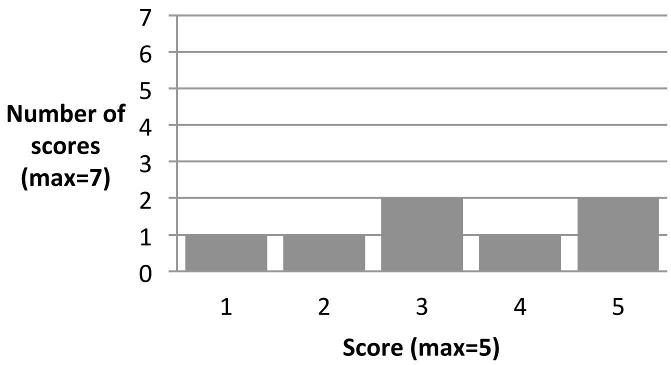

Comments from R1 Rankings: Inadequate evaluation of tool • Requires stairs, otherwise cheap and quick, working age stroke population but could be used for non-stroke, validity not tested • Small sample. Minimal change scores and SEM provided in paper • Only reliability tested •

**Measure ID**	**12**
**Name**	**Short Form of the Berg Balance Scale (SFBBS)**
**Reference**	Chou et al. Phys Ther. 2006
**Purpose**	To evaluate balance performance in people with stroke
**# of Items**	7
**Evaluation Parameters**	Categorical
**# of Categories**	3
**Graded Progression**	No

**Mean Round 1 Results**	
**Psychometric**	3.6
**Feasibility**	4.6
**Overall**	4.0

Psychometric Score Distribution

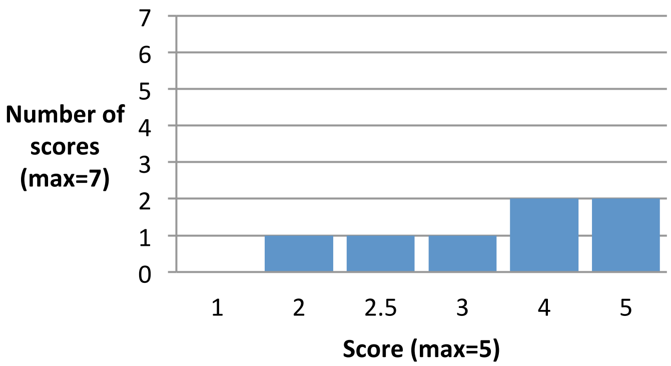

Feasibility Score Distribution

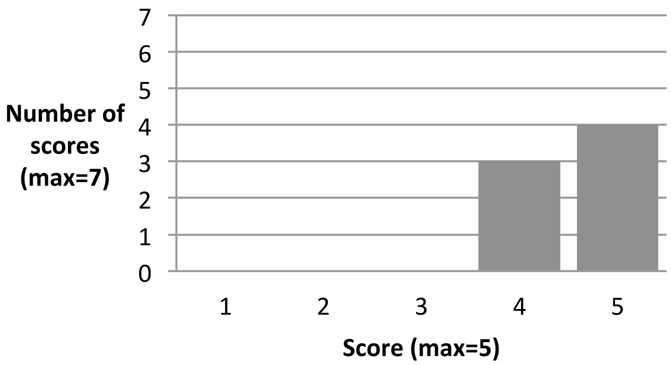

Comments from R1 Rankings: This short form would not take much time and is based on valid measures •

**Measure ID**	**13**
**Name**	**Short Berg Balance Scale**
**Reference**	Hohtari-Kivimaki et al. Aging-Clinical & Experimental Research. 2012
**Purpose**	To assess functional balance among community-dwelling aged with moderate or good physical functioning
**# of Items**	9
**Evaluation Parameters**	Categorical
**# of Categories**	5
**Graded Progression**	No

**Mean Round 1 Results**	
**Psychometric**	2.4
**Feasibility**	3.7
**Overall**	2.5

Psychometric Score Distribution

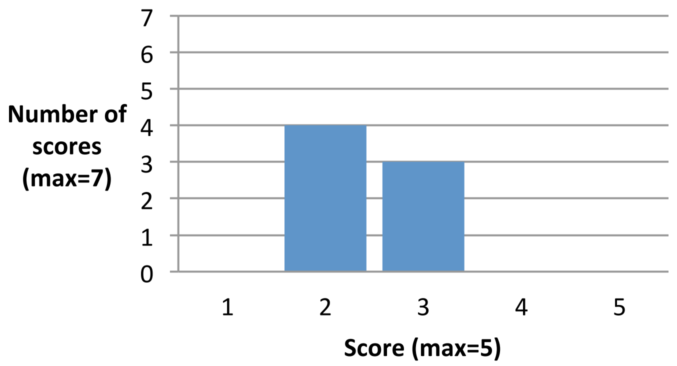

Feasibility Score Distribution

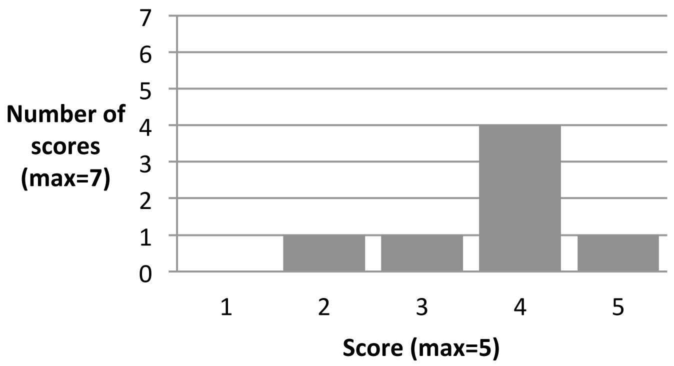

Comments from R1 Rankings: Insufficient evaluation; results mixed • Concurrent validity OK with force platform measures •

**Measure ID**	**14**
**Name**	**Brunel Balance Assessment (BBA)**
**Reference**	Tyson et al. Clin Rehabil. 2004
**Purpose**	To assess the effects of specific stroke physiotherapy interventions for balance disability post stroke
**# of Items**	12
**Evaluation Parameters**	Categorical
**# of Categories**	2
**Graded Progression**	Yes

**Mean Round 1 Results**	
**Psychometric**	2.4
**Feasibility**	3.7
**Overall**	2.3

Psychometric Score Distribution

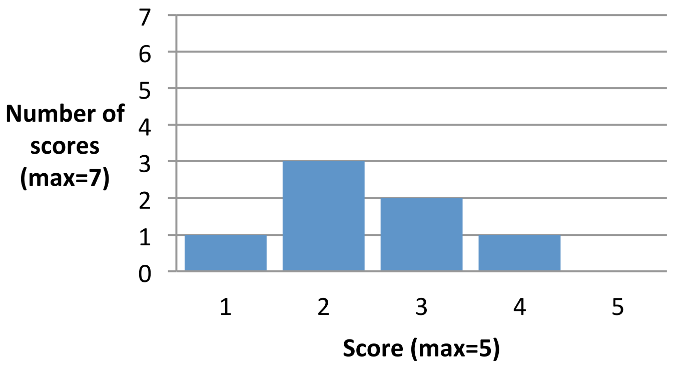

Feasibility Score Distribution

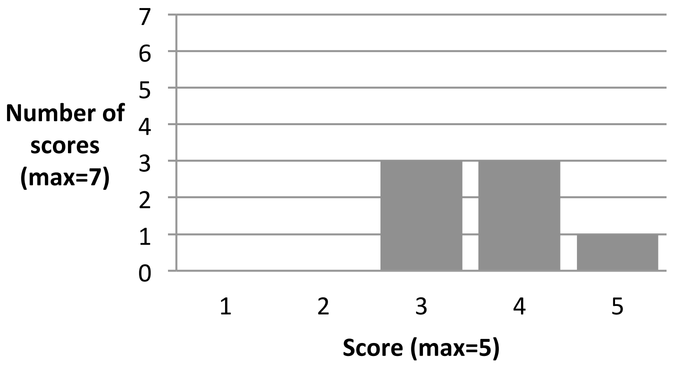

Comments from R1 Rankings: Insufficient evaluation; results mixed • Plinth, steps required, stroke population only, task force recommends only in stroke and not for research • One site, no replication •

**Measure ID**	**16**
**Name**	**Clinical Test of Sensory Interaction in Balance (CTSIB)**
**Reference**	??
**Purpose**	To assess the influence of sensory interaction on postural stability inthe standing patient with neurologic problems
**# of Items**	6
**Evaluation Parameters**	Suggests continuous (time) or categorical (subjective numeric ranking system for sway)
**# of Categories**	N/A
**Graded Progression**	No

**Mean Round 1 Results**	
**Psychometric**	2.4
**Feasibility**	2.6
**Overall**	2.2

Psychometric Score Distribution

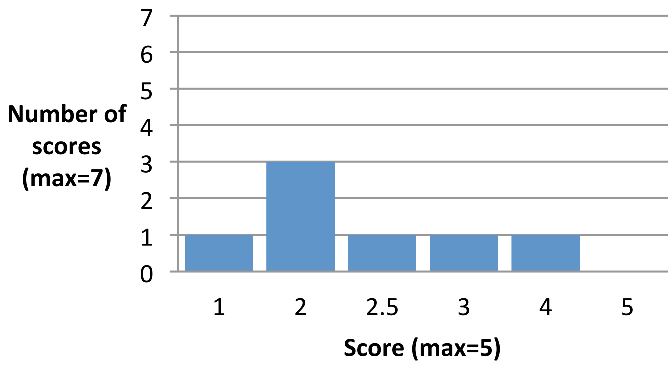

Feasibility Score Distribution

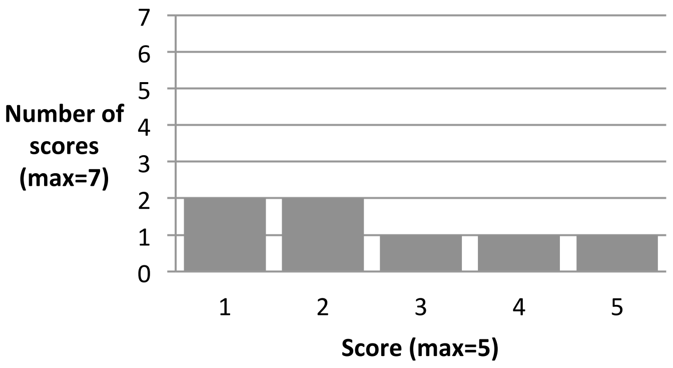

Comments from R1 Rankings: Potentially unsafe test to be conducted by untrained staff members • Measurement of sway is by observation. What data exists on performance is on small numbers only, but it has nice face validity. Not robust enough as it stands •

**Measure ID**	**17**
**Name**	**Community Balance and Mobility Scale (CB&M)**
**Reference**	Howe et al. Clin Rehabil. 2006
**Purpose**	To identify postural instability, evaluate change following intervention and inform rehabilitation team about balance and mobility status of ambulatory individuals with traumatic brain injury returning to community environment
**# of Items**	19
**Evaluation Parameters**	Categorical
**# of Categories**	6
**Graded Progression**	No

**Mean Round 1 Results**	
**Psychometric**	3.6
**Feasibility**	3.1
**Overall**	3.5

Psychometric Score Distribution

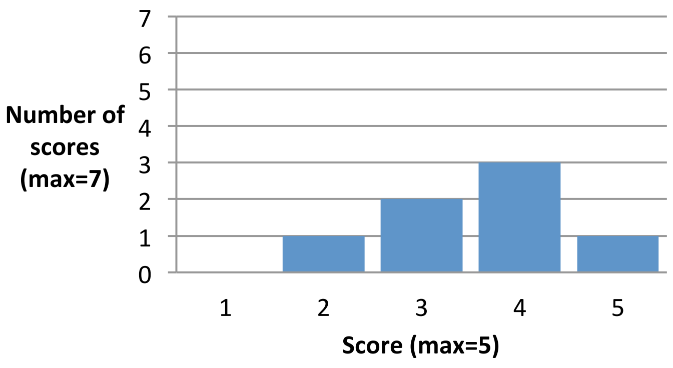

Feasibility Score Distribution

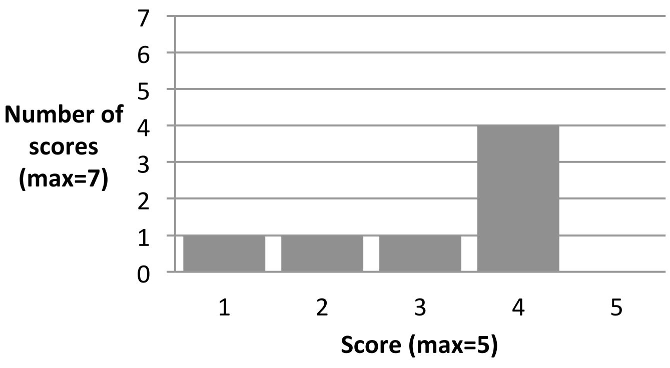

Comments from R1 Rankings: Excellent tool for TBI pts • May have a floor effect but this has not been conclusively demonstrated • Webased, time •

**Measure ID**	**18**
**Name**	**Dynamic Balance Assessment (DBA)**
**Reference**	Desai et al Phys Ther. 2010
**Purpose**	Not specified
**# of Items**	12
**Evaluation Parameters**	Categorical (continuous data collapsed into categories)
**# of Categories**	5
**Graded Progression**	No

**Mean Round 1 Results**	
**Psychometric**	2.2
**Feasibility**	2.3
**Overall**	2.2

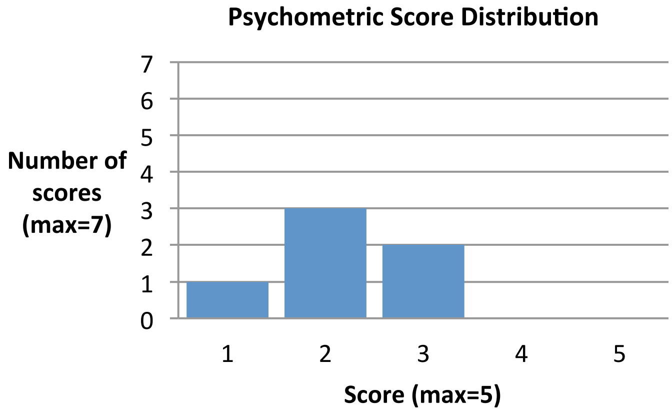

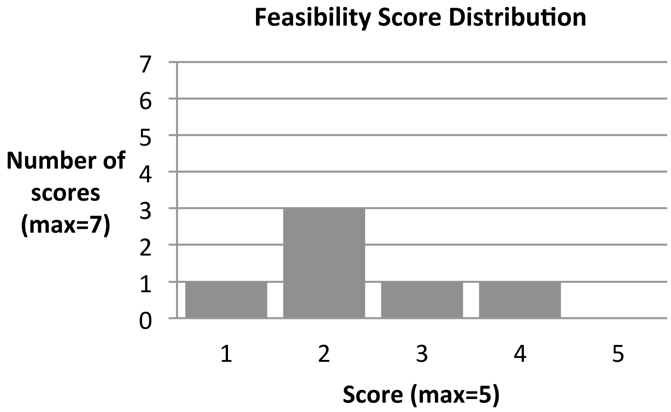

Comments from R1 Rankings: Insufficient evaluation; mixed results • Requires force platform, no cost given and nothing on training, only convergent validity given - community dwelling •

**Measure ID**	**19**
**Name**	**Dynamic Gait Index**
**Reference**	Shumway-Cook et al. Phys Ther. 1997
**Purpose**	To evaluate and document a patient's ability to modify gait in response to changing task demands
**# of Items**	8
**Evaluation Parameters**	Categorical
**# of Categories**	4
**Graded Progression**	No

**Mean Round 1 Results**	
**Psychometric**	3.9
**Feasibility**	3.2
**Overall**	3.0

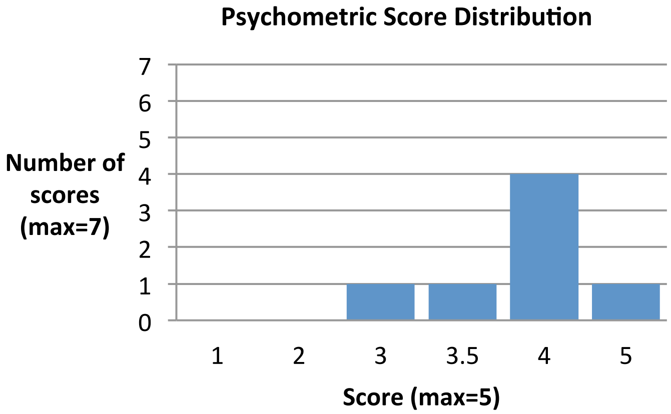

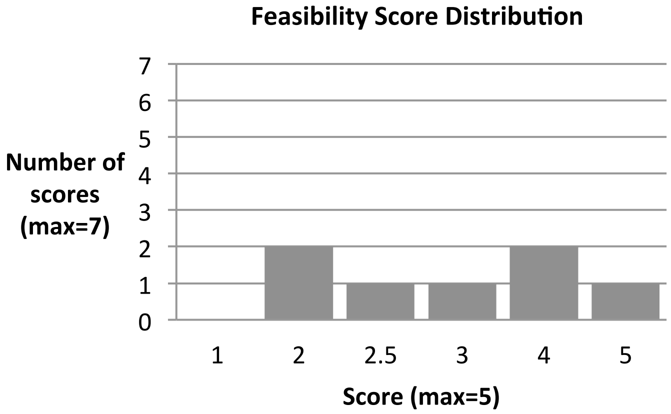

Comments from R1 Rankings: Cheap, takes less than 10 mins, but requires stairs, multiple LTC and comm dwelling, concerns about ceiling effects and poor responsiveness? • Same issue of time and training needed to conduct BERG • Requires person to be able to walk, so has a floor effect. The walk test requires a long walkway and hence may be problematic. Is a single snap shot of balance •

**Measure ID**	**20**
**Name**	**Four-item Dynamic Gait Index (4-DGI)**
**Reference**	Marchetti et al. Phys Ther. 2006
**Purpose**	To measure walking function in people with balance and vestibular disorders
**# of Items**	4
**Evaluation Parameters**	Categorical
**# of Categories**	4
**Graded Progression**	No

**Mean Round 1 Results**	
**Psychometric**	2.6
**Feasibility**	2.9
**Overall**	2.3

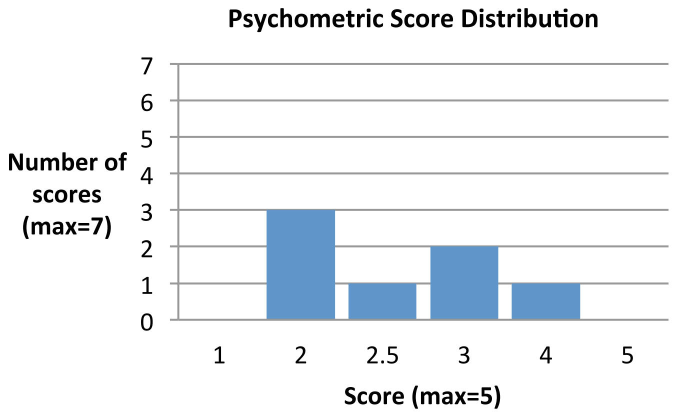

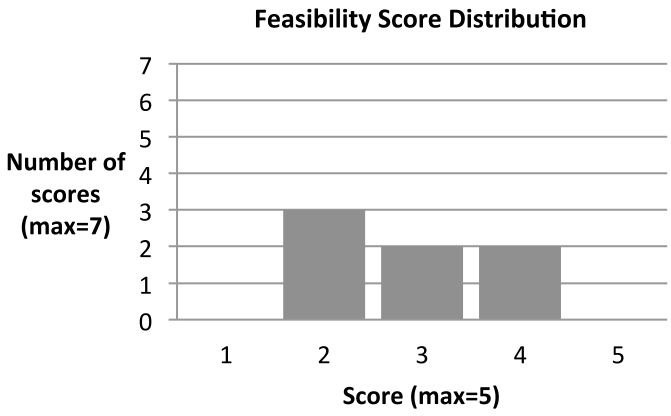

Comments from R1 Rankings: Easy to administer • Insufficient information on psychometric properties. Requires participants to be able to walk. Not clear if you can score zero if unable to walk •

**Measure ID**	**21**
**Name**	**Functional Gait Assessment (FGA)**
**Reference**	Wrisley et al. Phys Ther. 2004
**Purpose**	To assess postural stability during gait with higher-level tasks
**# of Items**	10
**Evaluation Parameters**	Categorical
**# of Categories**	4
**Graded Progression**	No

**Mean Round 1 Results**	
**Psychometric**	3.1
**Feasibility**	3.4
**Overall**	2.7

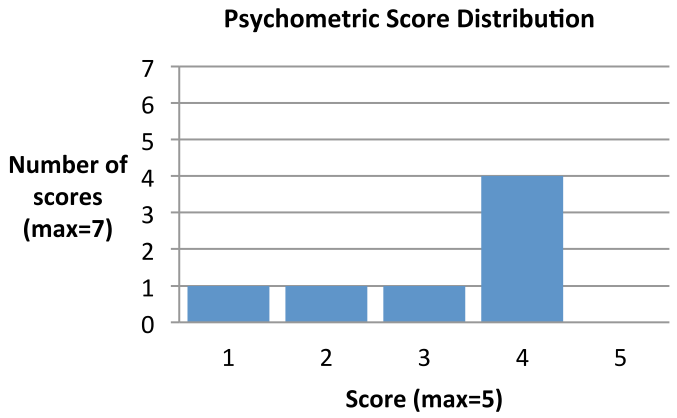

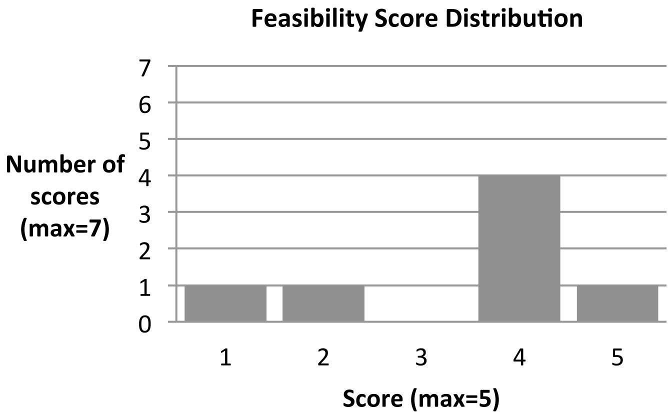

Comments from R1 Rankings: Well evaluated tool; multiple pops • Seems reasonable enough •

**Measure ID**	**22**
**Name**	**Five Times Sit-to-Stand Test (5-STS)**
**Reference**	Whitney et al. Phys Ther. 2005
**Purpose**	To measure balance dysfunction
**# of Items**	1
**Evaluation Parameters**	Continuous (time)
**# of Categories**	N/A
**Graded Progression**	No

**Mean Round 1 Results**	
**Psychometric**	3.3
**Feasibility**	3.9
**Overall**	2.8

Psychometric Score Distribution

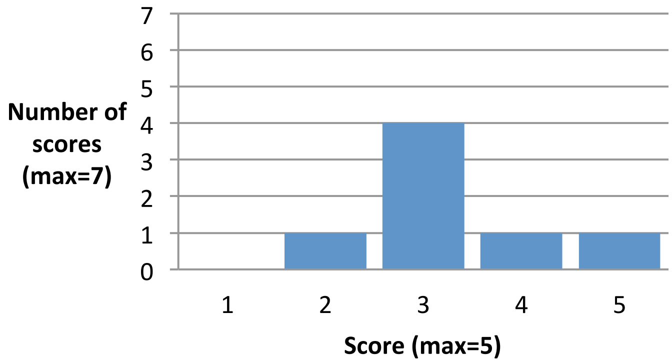

Feasibility Score Distribution

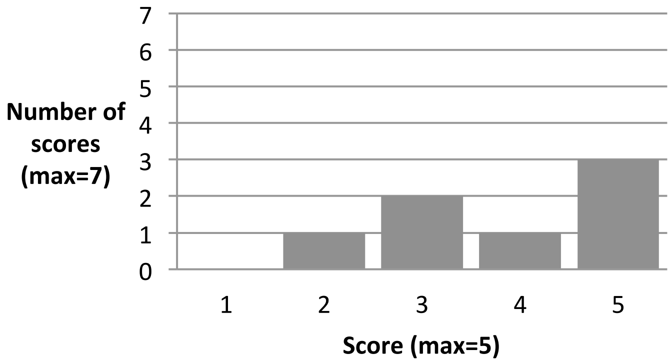

Comments from R1 Rankings: Better measure of LB strength • < 5 mins, cheap, no training, huge range of LTCs and OP, only one dimension of balance • I am not convinced that this is a measure that is specific to balance, and hence would not include it • Validity est. primarily for strength •

**Measure ID**	**23**
**Name**	**Four Square Step Test (FSST**
**Reference**	Dite and Temple. Arch Phys Med Rehabil. 2002
**Purpose**	Not specified
**# of Items**	1
**Evaluation Parameters**	Continuous (time)
**# of Categories**	N/A
**Graded Progression**	No

**Mean Round 1 Results**	
**Psychometric**	2.9
**Feasibility**	3.4
**Overall**	2.5

Psychometric Score Distribution

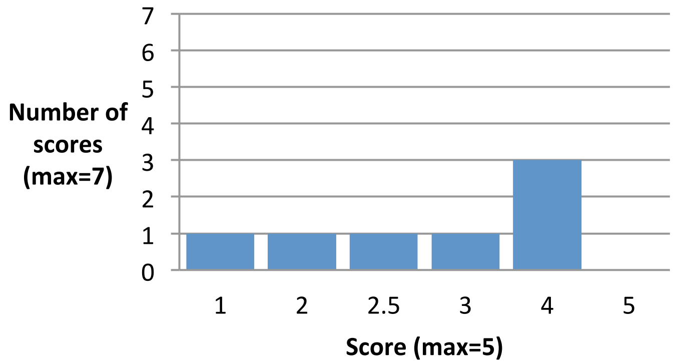

Feasibility Score Distribution

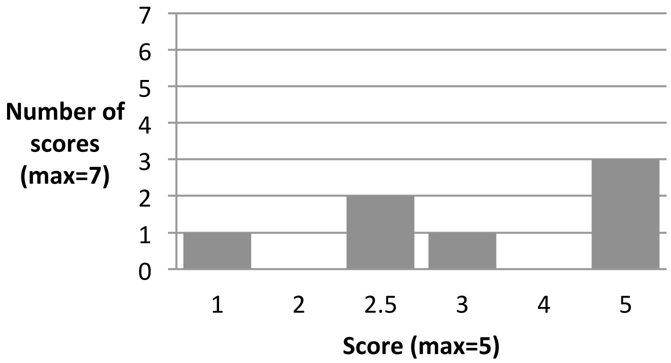

Comments from R1 Rankings: < 5 mins, cheap, but responsiveness in OP questionable? • Potentially unsafe test to be conducted by untrained staff members • Insufficient evidence of psychometric properties •

**Measure ID**	**24**		
**Name**	**Fullerton Advanced Balance (FAB) Scale**	**Mean Round 1 Results**	
**Reference**	Rose et al. Arch Phys Med Rehabil. 2006	**Psychometric**	2.9
**Purpose**	To identify balance problems of varying severity in functionally independent older adults and evaluate system(s) that might be contributing to balance problems	**Feasibility**	3.2
**# of Items**	10	**Overall**	2.7
**Evaluation Parameters**	Categorical		
**# of Categories**	5		
**Graded Progression**	No		

Psychometric Score Distribution

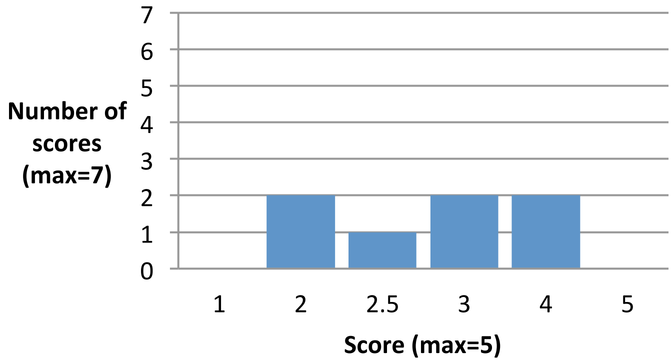

Feasibility Score Distribution

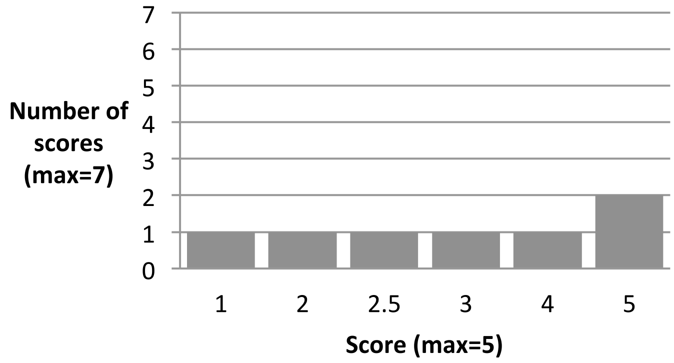

Comments from R1 Rankings: Insufficient evidence of psychometric properties • See value in individual items but test overall not well supported •

**Measure ID**	**25**		
**Name**	**Functional Reach Test**	**Mean Round 1 Results**	
**Reference**	Duncan et al. J Gerontol. 1990	**Psychometric**	3.6
**Purpose**	To assess anterior and posterior dynamic stability	**Feasibility**	4.0
**# of Items**	1	**Overall**	3.3
**Evaluation Parameters**	Continuous (distance)		
**# of Categories**	N/A		
**Graded Progression**	No		

Psychometric Score Distribution

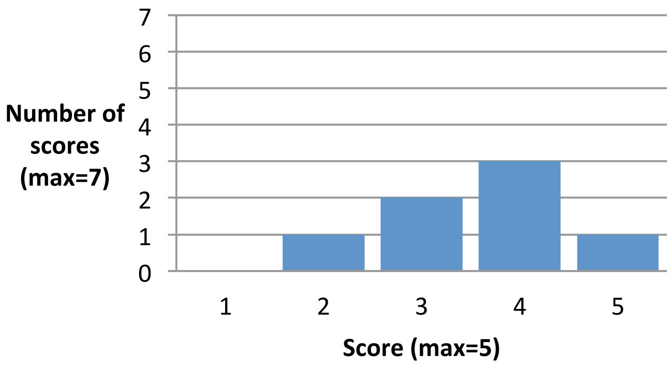

Feasibility Score Distribution

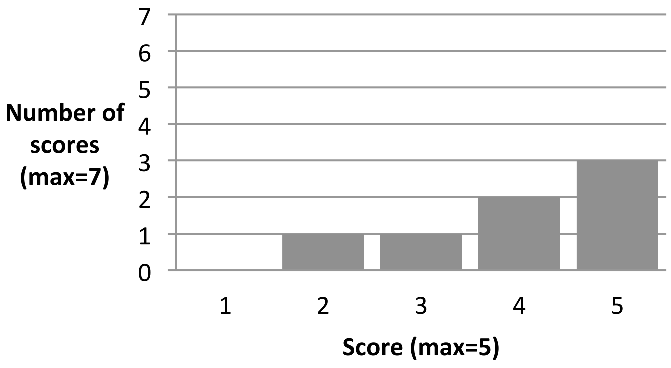

Comments from R1 Rankings: Screen only; very little use for determining best type of treatment • Should be considered but a practical limitation is the ability to stand and hold the shoulder to 90 degrees. Not always that easy in some populations (stroke), and is prone to non-completion in population surveys •

**Measure ID**	**26**
**Name**	**Multidirectional Reach Test**
**Reference**	Newton. J Gerontol A Biol Sci Med Sci. 2001
**Purpose**	To measure limits of stability in four reaching directions
**# of Items**	4
**Evaluation Parameters**	Continuous (distance)
**# of Categories**	N/A
**Graded Progression**	No

**Mean Round 1 Results**	
**Psychometric**	2.9
**Feasibility**	3.3
**Overall**	2.8

Psychometric Score Distribution

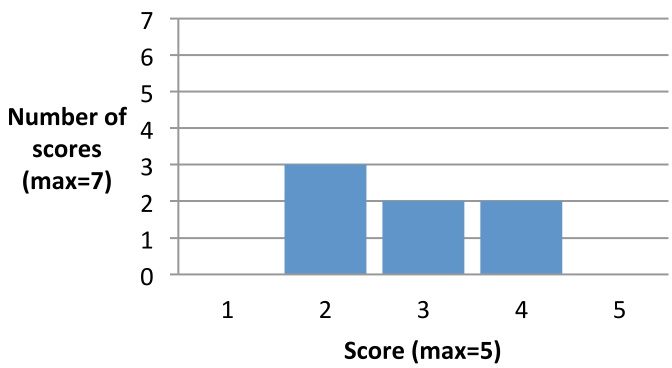

Feasibility Score Distribution

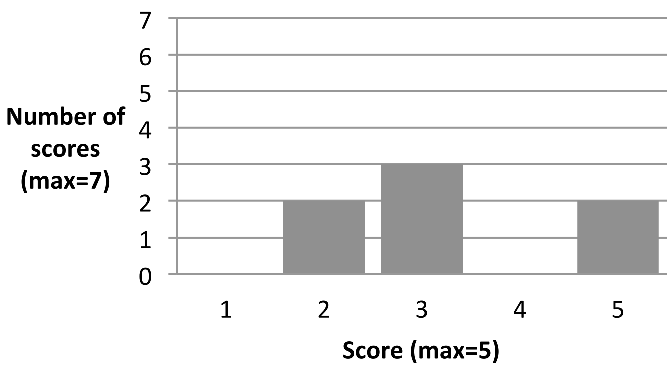

Comments from R1 Rankings: Inadequate evaluation of tool - lack of standardization re test administration • < 5 mins, poor reliability in OP, concerns about reaching and fear? Better than Functional Reach though! • Performance test requiring people to have good shoulder function and be able to stand. Likely to be difficult to use across a range of conditions •

**Measure ID**	**27**
**Name**	**Hierarchical Assessment of Balance and Mobility (HABAM)**
**Reference**	MacKnight and Rockwood. Age & Ageing 1995
**Purpose**	Not specified
**# of Items**	24
**Evaluation Parameters**	Categorical
**# of Categories**	2
**Graded Progression**	Yes

**Mean Round 1 Results**	
**Psychometric**	2.4
**Feasibility**	3.2
**Overall**	2.3

Psychometric Score Distribution

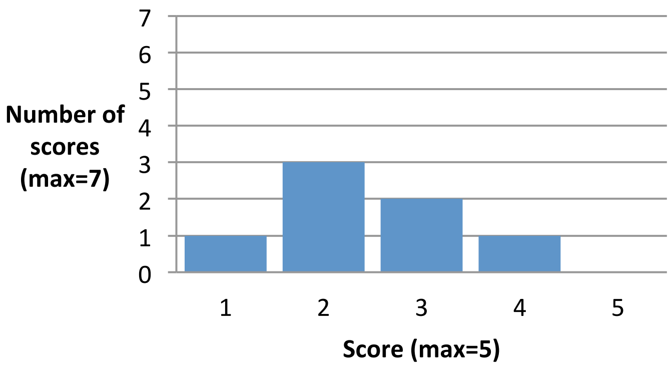

Feasibility Score Distribution

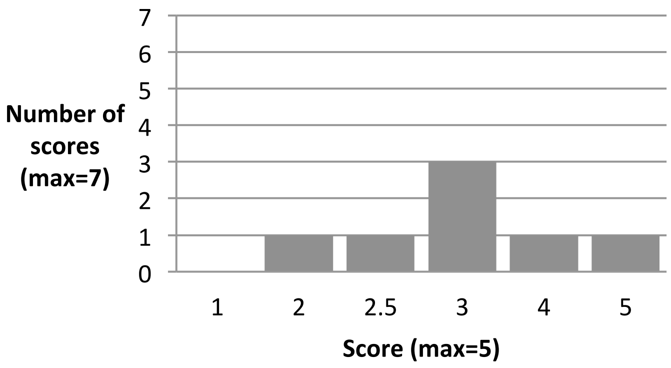

Comments from R1 Rankings: Quick, cheap and shows progression but concerns about ceiling effects in higher functioning OP. Used in hospital settings so far • Too many items • I am surprised that this really neat little measure does not have more psychometric data to support its performance •

**Measure ID**	**28**
**Name**	**Limits of Stability Test (LOS)**
**Reference**	Clark et al. Arch Phys Med Rehabil. 1997
**Purpose**	To assess multiple indices of dynamic balance performance by evaluating individual's ability to volitionally move the center of gravity to 8 predetermined positions
**# of Items**	8
**Evaluation Parameters**	Continuous (center of gravity velocity, excursion, endpoint, directional control)
**# of Categories**	N/A
**Graded Progression**	No

**Mean Round 1 Results**	
**Psychometric**	2.3
**Feasibility**	1.7
**Overall**	1.7

Psychometric Score Distribution

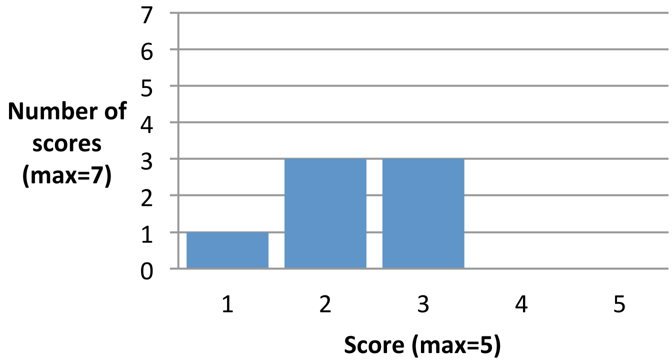

Feasibility Score Distribution

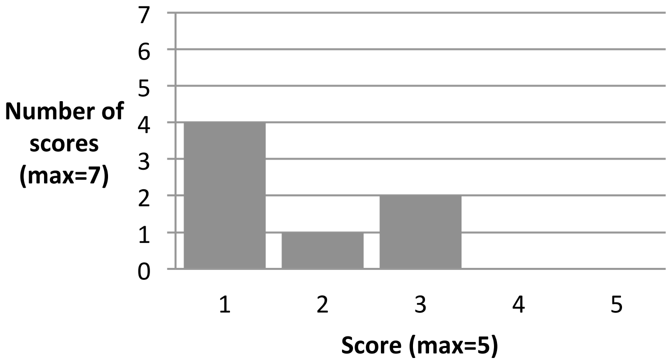

Comments from R1 Rankings: Quite a good test, but with inadequate evidence of psychometric properties •

**Measure ID**	**31**
**Name**	**Performance Oriented Mobility Assessment (POMA)**
**Reference**	Tinetti. J Am Geriatr Soc. 1986
**Purpose**	To practically assess performance-oriented mobility tasks that incorporates useful features of both disease-oriented and gait analytic approaches
**# of Items**	Balance- 13, Gait- 9
**Evaluation Parameters**	Categorical
**# of Categories**	3 for balance item and 2 for gait items
**Graded Progression**	No

**Mean Round 1 Results**	
**Psychometric**	3.6
**Feasibility**	3.7
**Overall**	3.3

Psychometric Score Distribution

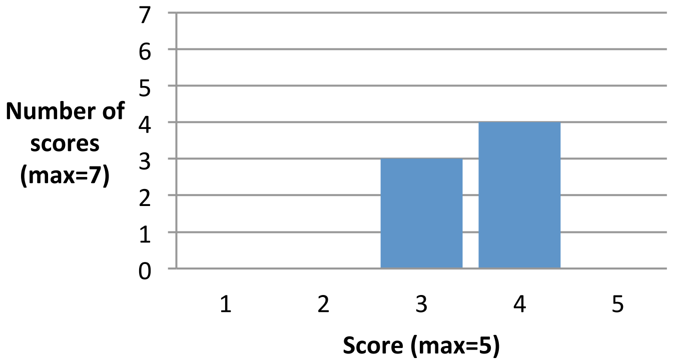

Feasibility Score Distribution

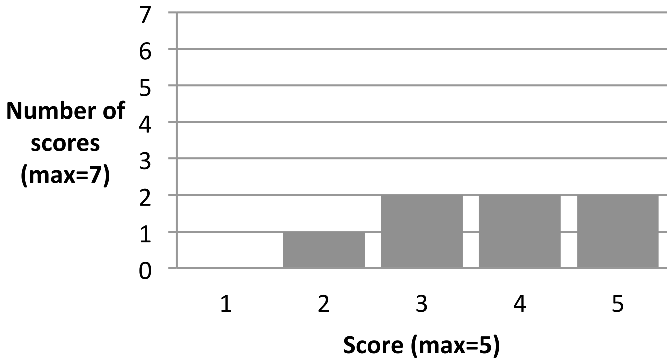

Comments from R1 Rankings: Can take up to 30 mins, wide range of LTCs and OP, cheap • Training required to conduct properly. Takes up to 15 minutes to compete • There is a lot of data on this measure that is not picked up in the review. It has a very solid epidemiological base, and is a good simple measure • Sub-optimal inclusion of balance measures, requires clinical acumen • Questionable for higher functioning adults • Ceiling effects, aimed at elderly, no responses or sensory conditions •

**Measure ID**	**33**
**Name**	**Postural Assessment Scale for Stroke Patients (PASS)**
**Reference**	Benain et al. Stroke. 1999
**Purpose**	To assess and monitor postural control after stroke; to assess subject performance
**# of Items**	14
**Evaluation Parameters**	Categorical only
**# of Categories**	4
**Graded Progression**	No

**Mean Round 1 Results**	
**Psychometric**	3.0
**Feasibility**	3.4
**Overall**	2.7

Psychometric Score Distribution

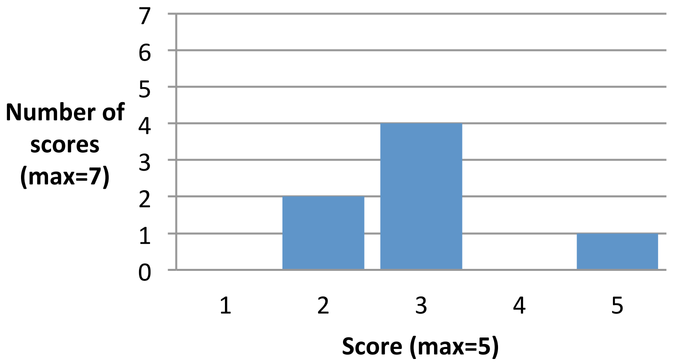

Feasibility Score Distribution

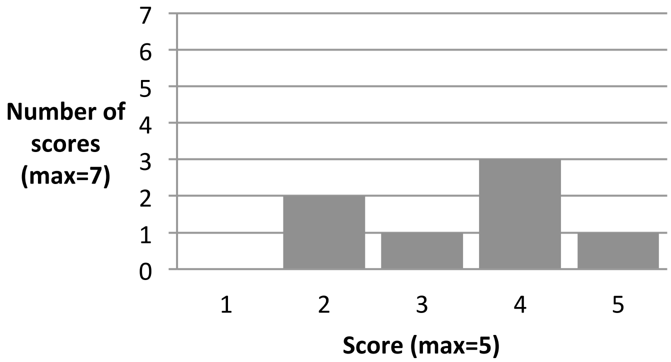

Comments from R1 Rankings: • Useful measure of progress in first 3 mos post-stroke • Has some evidence of psychmetric adequacy but is limited to stroke populations • Small study for psychometric assessment • Not general for all elderly •

**Measure ID**	**34**
**Name**	**Short Form of Postural Assessment Scale for Stroke Patients (SFPASS)**
**Reference**	Chien et al. Neurorehabil Neur Repair. 2007
**Purpose**	To measure balance function in people with stroke
**# of Items**	5
**Evaluation Parameters**	Categorical
**# of Categories**	3
**Graded Progression**	No

**Mean Round 1 Results**	
**Psychometric**	3.7
**Feasibility**	4.2
**Overall**	3.2

Psychometric Score Distribution

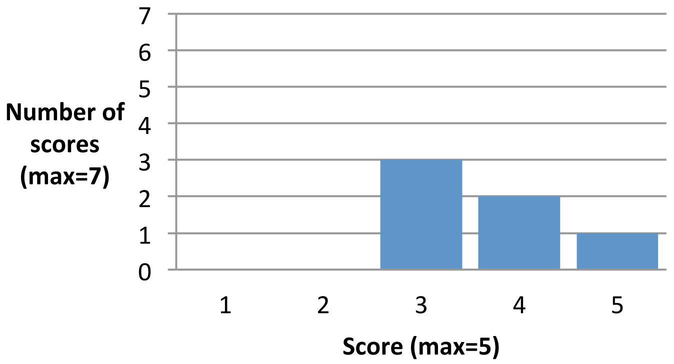

Feasibility Score Distribution

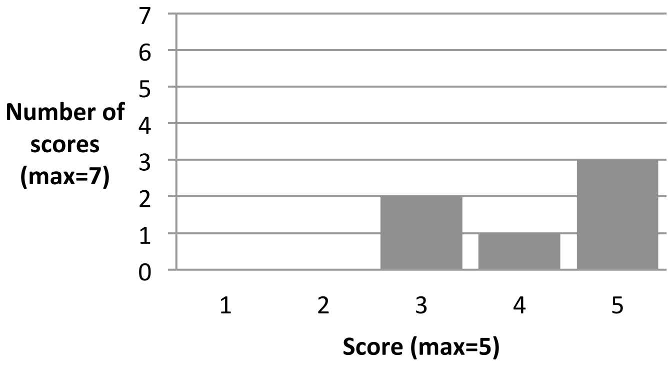

Comments from R1 Rankings: Could be used in conjunction with PASS to monitor change during treatment phase • Stroke specific, quick and cheap, can be used with non stroke but not done so yet :(• Psychometric data limited to stroke, where it is good. Not sure if you are intending to give disease specific recommendations, if so include. If not exclude •

**Measure ID**	**35**
**Name**	**Pull/ Retropulsion Test**
**Reference**	Visser et al. Arch Phys Med Rehabil. 2003
**Purpose**	To assess the ability to maintain balance
**# of Items**	1
**Evaluation Parameters**	Categorical
**# of Categories**	4
**Graded Progression**	No

**Mean Round 1 Results**	
**Psychometric**	2.5
**Feasibility**	3.7
**Overall**	2.8

Psychometric Score Distribution

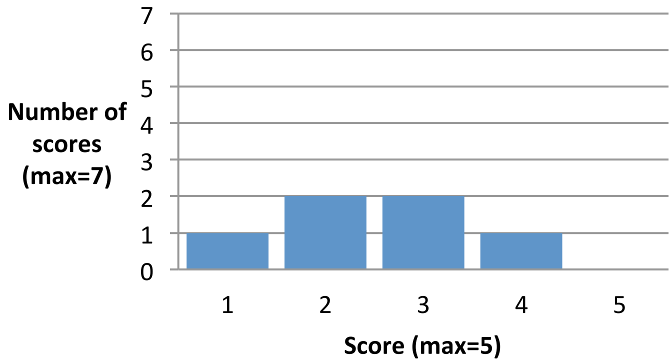

Feasibility Score Distribution

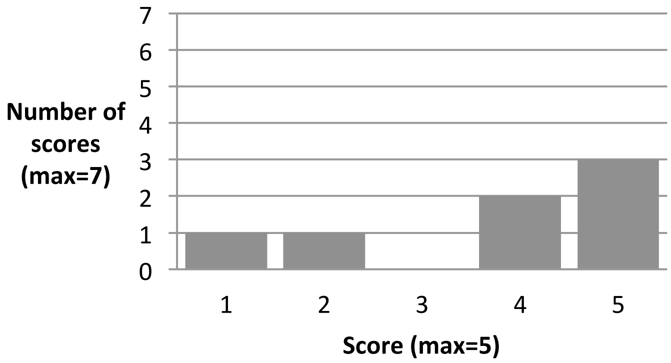

Comments from R1 Rankings: Quick, but limited info on balance, no test retest • Potentially unsafe test to be conducted by untrained staff members • Limited to Parkinson’s only, pull test has quite a lot of difficulties in administration • Important component •

**Measure ID**	**36**
**Name**	**Push and Release Test**
**Reference**	Jacobs et al. J Neurol. 2006
**Purpose**	To reliably assess postural stability with sensitivity to fall history and low balance confidence in Parkinson's Disease
**# of Items**	1
**Evaluation Parameters**	Categorical
**# of Categories**	5
**Graded Progression**	No

**Mean Round 1 Results**	
**Psychometric**	2.7
**Feasibility**	3.7
**Overall**	3.3

Psychometric Score Distribution

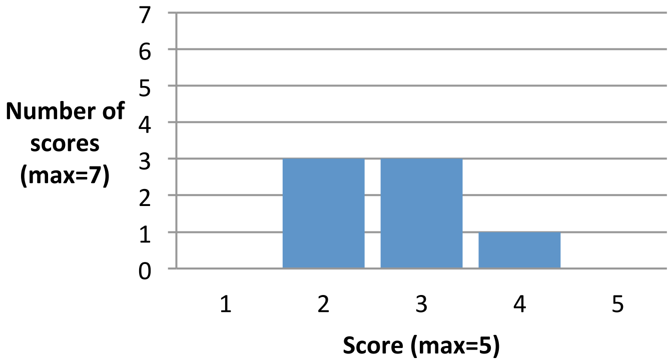

Feasibility Score Distribution

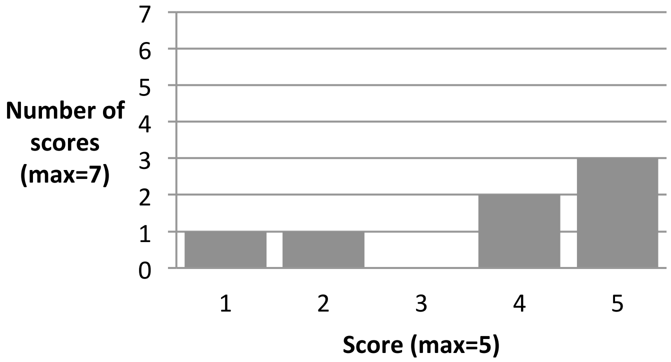

Comments from R1 Rankings: Potentially unsafe test to be conducted by untrained staff members • Limited to Parkinson’s, limited evidence on psychometric tests, likely to be hard to implement in a standardized reliable way • Conflict; important component •

**Measure ID**	**38**
**Name**	**Sensory Organization Test (SOT)**
**Reference**	Ford-Smith et al. Arch Phys Med Rehabil. 1995
**Purpose**	To assess ability to make effective use of visual, vestibular, and proprioceptive inputs separately and the ability to suppress inaccurate sensory information
**# of Items**	6
**Evaluation Parameters**	Continuous (2 outcomes per condition)
**# of Categories**	N/A
**Graded Progression**	No

**Mean Round 1 Results**	
**Psychometric**	2.3
**Feasibility**	1.7
**Overall**	1.7

Psychometric Score Distribution

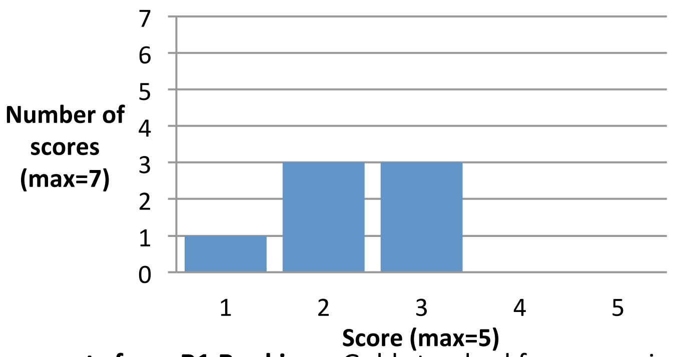

Feasibility Score Distribution

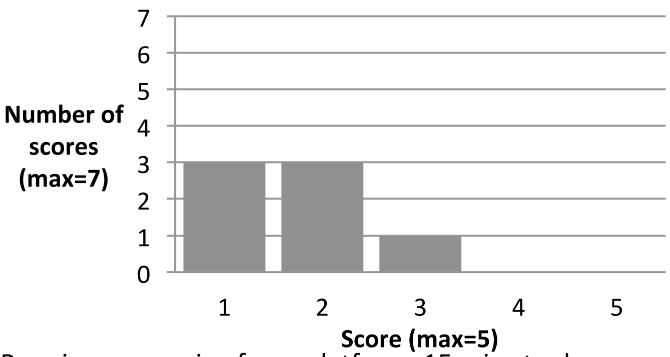

Comments from R1 Rankings: Gold standard for measuring SR & I • Requires expensive force platform, 15 mins to do, very expensive, test retest not brilliant nothing on reliability • Insensitive to general balance problems, more a diagnostic tool for specific balance problems. Expensive and requiring force platform, 15 mins to do, very expensive, test retest not brilliant nothing on reliability • What about CTSIB? Limited scope •

**Measure ID**	**40**
**Name**	**Short Physical Performance Battery (SPPB)**
**Reference**	Guralnik et al. J Gerontol. 1994
**Purpose**	To assess lower extremity function
**# of Items**	6
**Evaluation Parameters**	Standing and walking items = categorical; Rise from sitting item = continuous (time)
**# of Categories**	Timed standing: side-by-side stand = 2, semi-tandem = 5, tandem = 3. Walking item: 5 categories depending on time
**Graded Progression**	Standing and rise from sitting items were graded

**Mean Round 1 Results**	
**Psychometric**	3.4
**Feasibility**	4.6
**Overall**	3.2

Psychometric Score Distribution

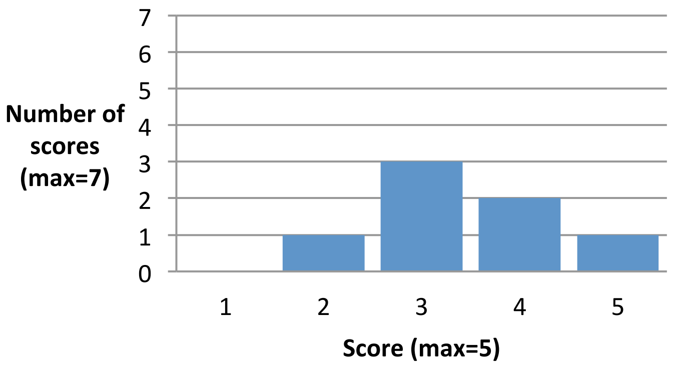

Feasibility Score Distribution

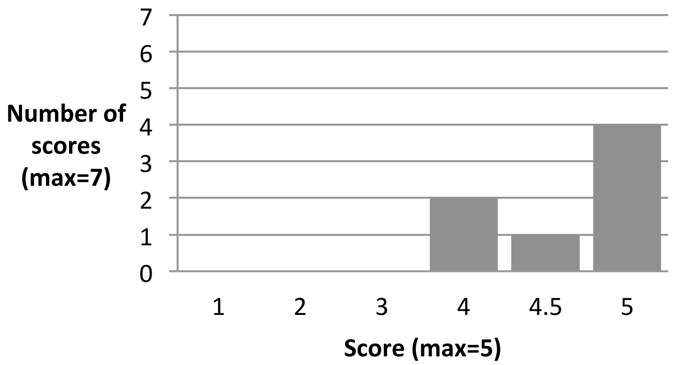

Comments from R1 Rankings: Feasibility of use enhanced by training video available for lay people to conduct the test safely in client homes • A good measure which has a mass of data to support various aspects of validity (not included in the review). Simple to implement and use, and reasonably sensitive. Already being used in clinical trials. Should be considered • Intended to predict disability, mortality, nursing home admission • Needs postural response and sensory conditions and dual tasks •

**Measure ID**	**41**
**Name**	**Side-Step Test**
**Reference**	Fujisawa et al. Clin Rehabil. 2006
**Purpose**	To assess dynamic standing balance in the frontal plane
**# of Items**	1
**Evaluation Parameters**	Continuous (distance)
**# of Categories**	N/A
**Graded Progression**	No

**Mean Round 1 Results**	
**Psychometric**	2.7
**Feasibility**	3.3
**Overall**	2.6

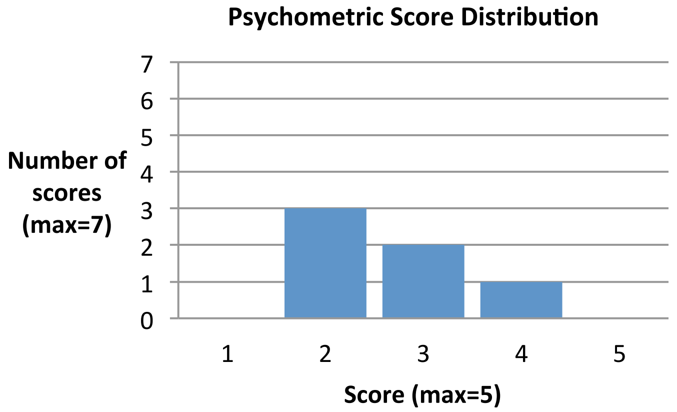

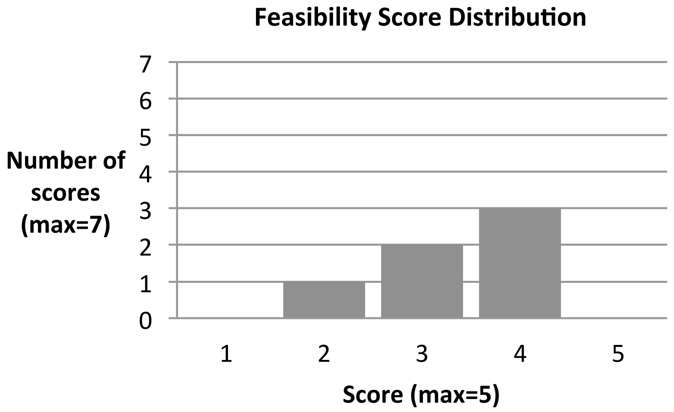

Comments from R1 Rankings: Better tools to evaluate balance in post-stroke population • Inadequate evidence for psychometric qualities •

**Measure ID**	**43**
**Name**	**Single leg Stance Test**
**Reference**	Bohannon. Topics Geri Rehabil. 2006
**Purpose**	To quantify standing balance
**# of Items**	1 or 2 (if one leg or both legs tested)
**Evaluation Parameters**	Continuous (time)
**# of Categories**	N/A
**Graded Progression**	No

**Mean Round 1 Results**	
**Psychometric**	2.4
**Feasibility**	3.9
**Overall**	2.7

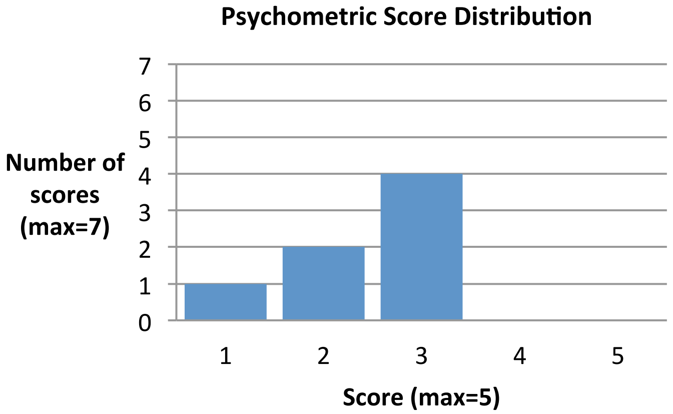

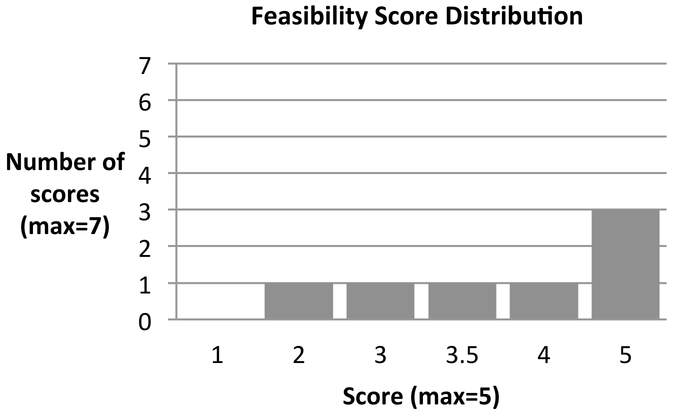

Comments from R1 Rankings: very quick, only one aspect of balance, floor effects as many OP cannot stand on 1 leg, little on psychom prop • Easy to administer • Inadequate evidence for psychometric qualities, limited to Parkinson's only •Impractical for elderly •

**Measure ID**	**44**
**Name**	**Spring Scale Test (SST)**
**Reference**	DePasquale and Toscano. J Geri Phys Ther. 2009
**Purpose**	To assess and quantify effective limits of anterior-posterior stepping for the purposes of fall risk assessment
**# of Items**	2
**Evaluation Parameters**	Continuous (% body weight)
**# of Categories**	N/A
**Graded Progression**	Yes

**Mean Round 1 Results**	
**Psychometric**	2.6
**Feasibility**	2.7
**Overall**	2.2

Psychometric Score Distribution

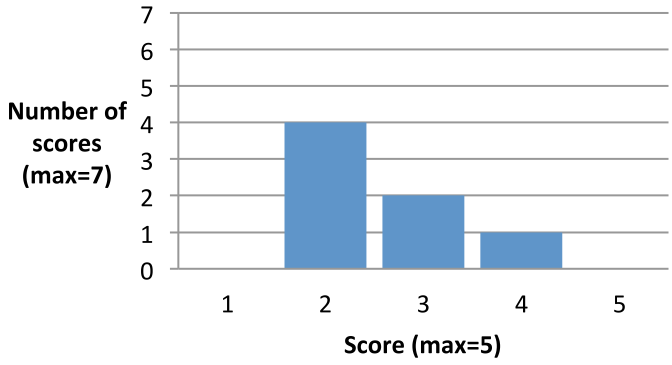

Feasibility Score Distribution

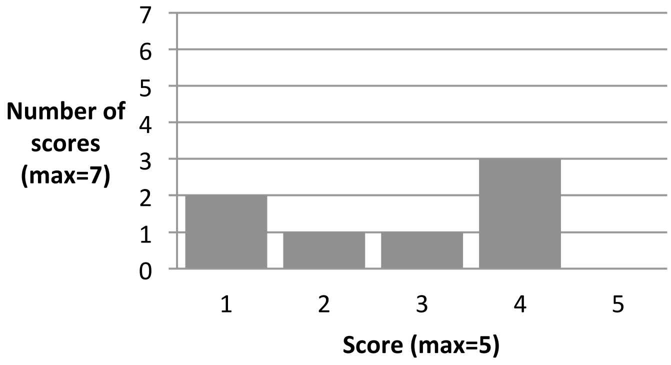

Comments from R1 Rankings: Equipment cost of $160. training not mentioned •

**Measure ID**	**47**
**Name**	**Step Test (ST)**
**Reference**	Hill et al. Physiotherapy Canada. 1996
**Purpose**	To meet the need for a clinically useful test of balance that incorporates dynamic single limb stance
**# of Items**	6
**Evaluation Parameters**	Continuous (number of steps up to 7.5 cm in 15 and 30 s and 15 cm in 15 s on each leg)
**# of Categories**	N/A
**Graded Progression**	No

**Mean Round 1 Results**	
**Psychometric**	2.7
**Feasibility**	3.8
**Overall**	2.6

Psychometric Score Distribution

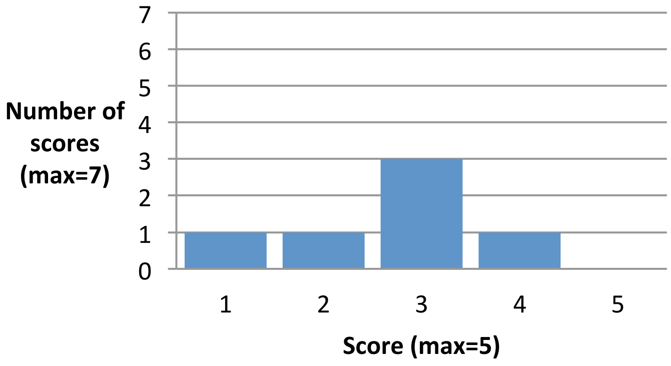

Feasibility Score Distribution

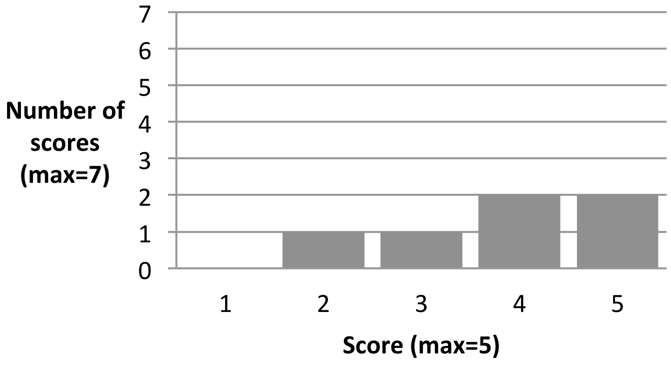

Comments from R1 Rankings: <5 mins, needs a step, will be floor effects?, psych prop in stroke patients but also used in OP • Limited practical application compared to more multidimensional measures • See some value in this task more generally •

**Measure ID**	**48**
**Name**	**Tandem Stance**
**Reference**	Hile et al. Phys Ther 2012
**Purpose**	To assess postural stability by narrowing the base of support
**# of Items**	2
**Evaluation Parameters**	Continuous (time)
**# of Categories**	N/A
**Graded Progression**	No

**Mean Round 1 Results**	
**Psychometric**	1.9
**Feasibility**	3.6
**Overall**	2.2

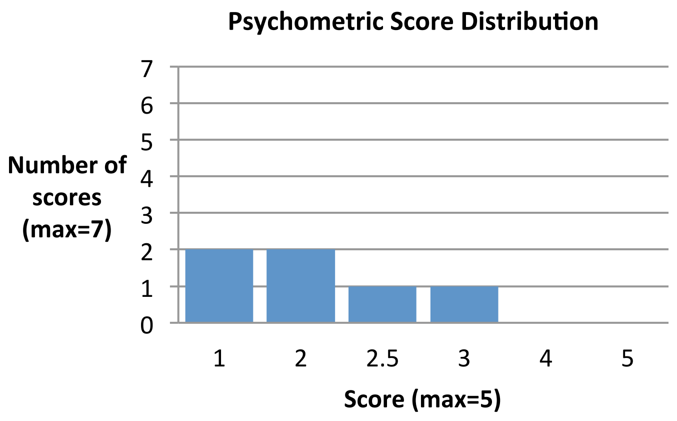

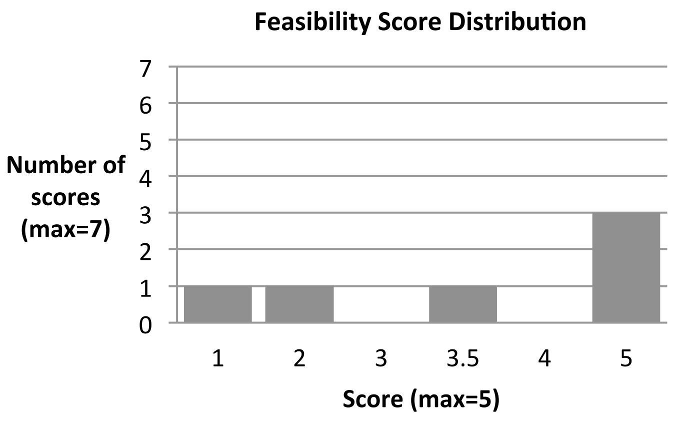

Comments from R1 Rankings: Easy to add hold time to existing tandum stance measure • See some value in this task more generally •

**Measure ID**	**50**
**Name**	**Expanded Timed Up-and-Go Test (ETUG)**
**Reference**	Botolfsen et al. Phys Res Int. 2008
**Purpose**	To address shortcomings of the Get-up-and-Go and TUG tests
**# of Items**	5
**Evaluation Parameters**	Continuous (time)
**# of Categories**	N/A
**Graded Progression**	No

**Mean Round 1 Results**	
**Psychometric**	3.0
**Feasibility**	4.0
**Overall**	3.3

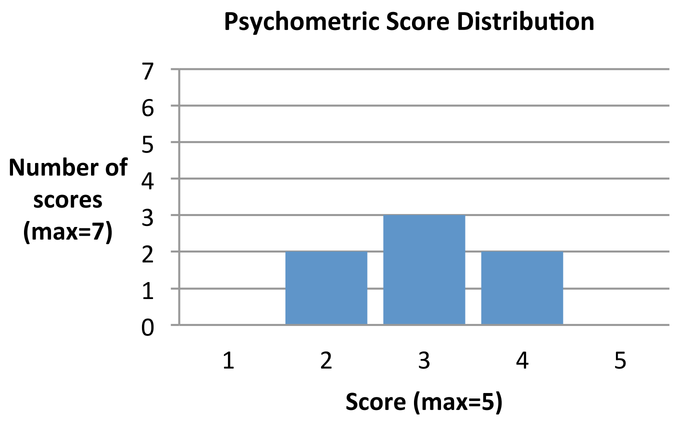

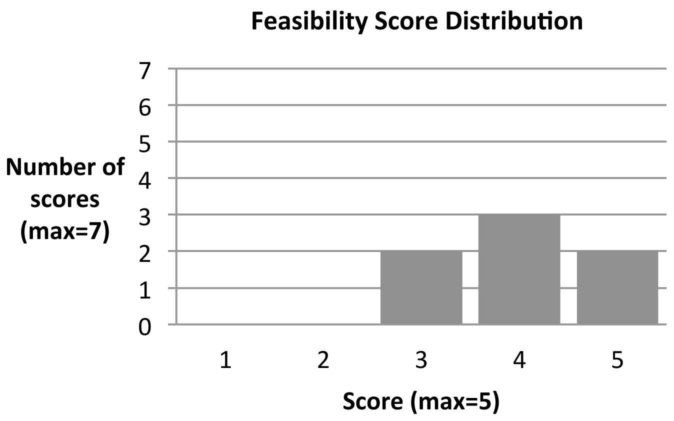

Comments from R1 Rankings: Evaluates more dimensions of balance but tool needs further testing on more samples • more info on balance than TUG, but limited psych prop, •

Round 1 Results: Discuss (n=39)

**Measure ID**	**51**
**Name**	**TURN180**
**Reference**	Simpson et al. Physiotherapy. 2002
**Purpose**	To be a simple, clinically useful test of dynamic postural control in frail elderly people
**# of Items**	2
**Evaluation Parameters**	Continuous (counting number of steps)
**# of Categories**	N/A
**Graded Progression**	No

**Mean Round 1 Results**	
**Psychometric**	2.4
**Feasibility**	3.9
**Overall**	2.7

Psychometric Score Distribution

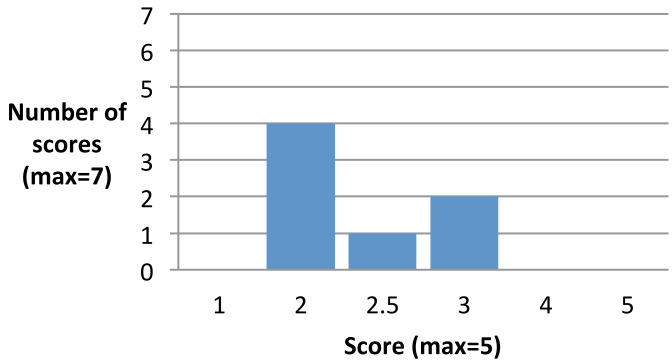

Feasibility Score Distribution

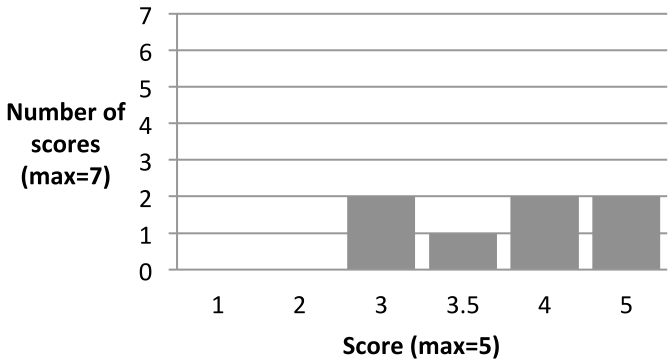

Comments from R1 Rankings: Cheap, quick, turning only, may have ceiling effect as only on frail OP ? Responsiveness • 180 degree turns with supports in place may be valuable measure to add to other balance measures for frail older adults • Limited components •

**Measure ID**	**52**
**Name**	**Unified Balance Scale**
**Reference**	La Porta et al. J Rehabil Med. 2011
**Purpose**	To be a single tool with proven measurement properties, allowing the measurement of balance “from bed to community” regardless of the etiology of the neurological lesion causing the loss of balance
**# of Items**	27
**Evaluation Parameters**	Categorical
**# of Categories**	2-5, depending on question
**Graded Progression**	No

**Mean Round 1 Results**	
**Psychometric**	3.1
**Feasibility**	3.2
**Overall**	3.0

Psychometric Score Distribution

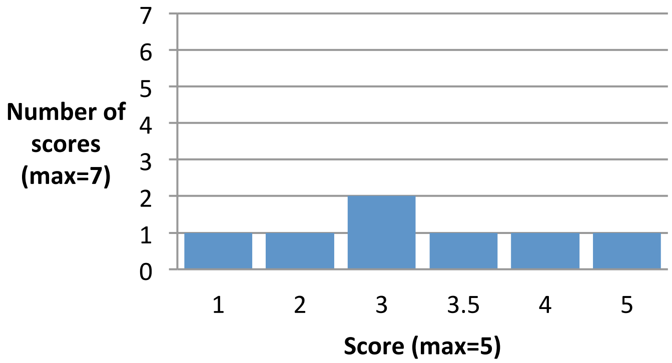

Feasibility Score Distribution

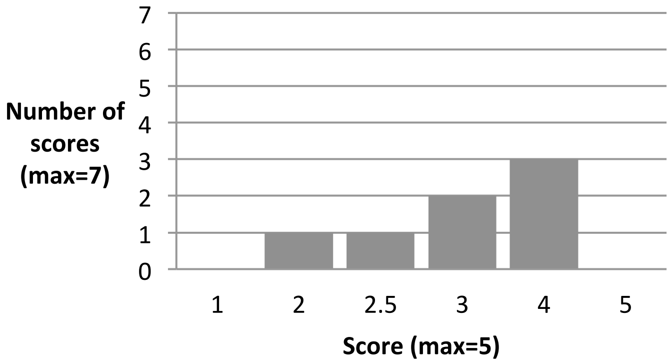

Comments from R1 Rankings: Further work needed to reduce the number of items to reduce the overall time needed to administer. But a good start on identifying the most relevant measures to be included •

**Measure ID**	**55**		
**Name**	**High Level Mobility Assessment Tool (HiMAT)**	**Mean Round 1 Results**	
**Reference**	Williams et al. Brain Inj. 2005	**Psychometric**	3.0
**Purpose**	To assess people with high level mobility and balance problems	**Feasibility**	3.1
**# of Items**	9 tasks, 13 items assessed	**Overall**	2.0
**Evaluation Parameters**	Categorical		
**# of Categories**	5		
**Graded Progression**	No		

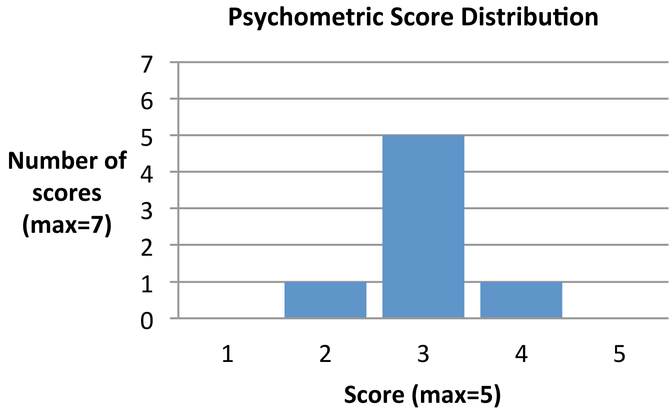

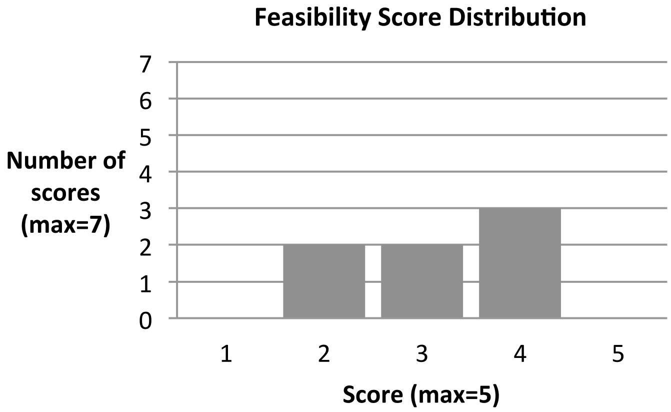

Comments from R1 Rankings: Cheap, quick but will be floor effects in less well functioning used in TBI • For use with younger adults with TBI • Not for elderly • TBI specific •

**Measure ID**	**11**
**Name**	**Berg Balance Scale (BBS)**
**Reference**	Berg et al. Physiotherapy Canada. 1989
**Purpose**	To measure balance in healthy individuals
**# of Items**	14
**Evaluation Parameters**	Categorical
**# of Categories**	5
**Graded Progression**	No

**Mean Round 1 Results**	
**Psychometric**	4.3
**Feasibility**	3.7
**Overall**	3.8

Psychometric Score Distribution

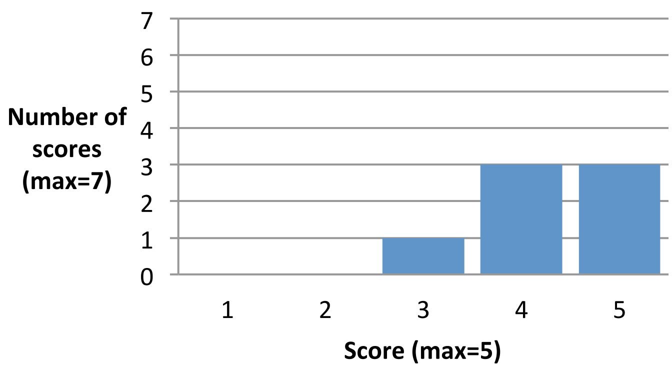

Feasibility Score Distribution

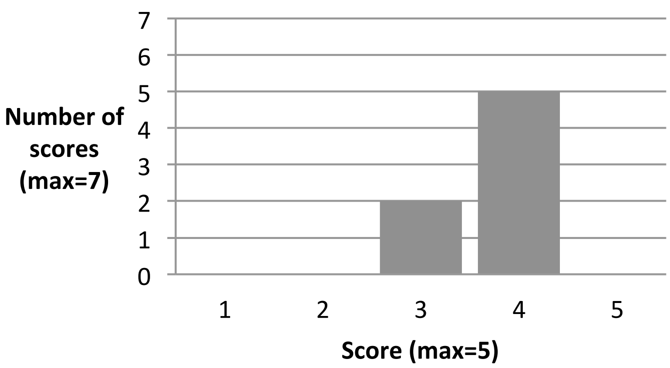

Comments from R1 Rankings: 15-20 mins makes this less feasible on large scale, used in wide variety of LTCs and trials, cheap and most physios have trained using it • BERG test needs specific training and takes time to complete • Most widely used and researched measure. Long assessment, ordinal scale, ceiling effect •

**Measure ID**	**49**
**Name**	**Timed Up-and-Go Test (TUG)**
**Reference**	Podsiadlo et al. J Am Geriatr Soc. 1991
**Purpose**	To quickly assess basic mobility skills
**# of Items**	1
**Evaluation Parameters**	Continuous (time)
**# of Categories**	N/A
**Graded Progression**	No

**Mean Round 1 Results**	
**Psychometric**	3.0
**Feasibility**	3.1
**Overall**	2.0

Psychometric Score Distribution

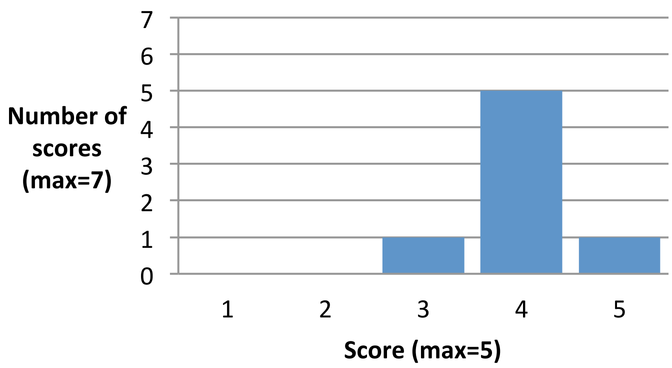

Feasibility Score Distribution

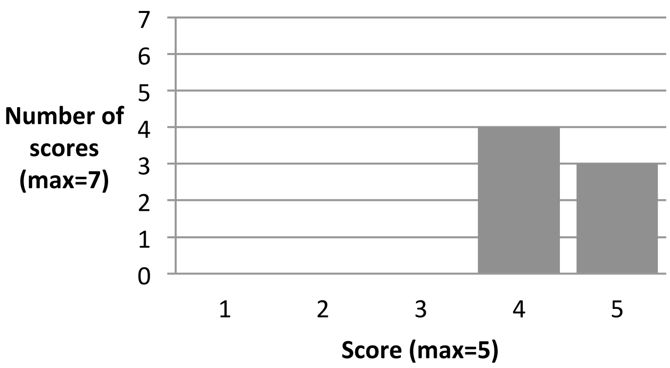

Comments from R1 Rankings: Screening tool only - inadequate measure of multiple dimensions of balance • Too short gait •

Round 1 Results: Exclude (n=15)

**Measure ID**	**1**
**Name**	**Activity-based Balance Level Evaluation (ABLE) Scale**
**Reference**	Ardolino et al. Phys Ther. 2012
**Purpose**	To assess changes in balance across the full spectrum of recovery in the spinal cord injury population
**# of Items**	28
**Evaluation Parameters**	Categorical
**# of Categories**	5
**Graded Progression**	No

**Mean Round 1 Results**	
**Psychometric**	1.9
**Feasibility**	2.3
**Overall**	1.7

Psychometric Score Distribution

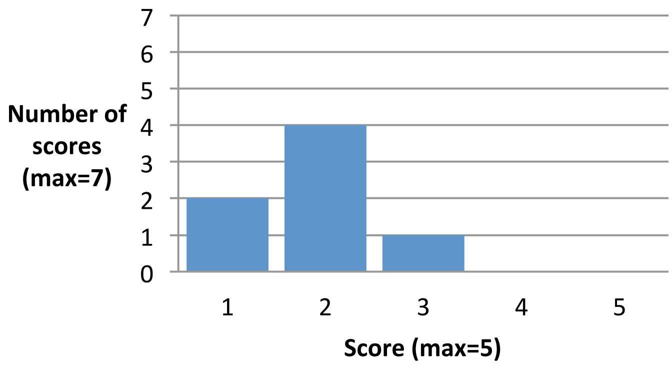

Feasibility Score Distribution

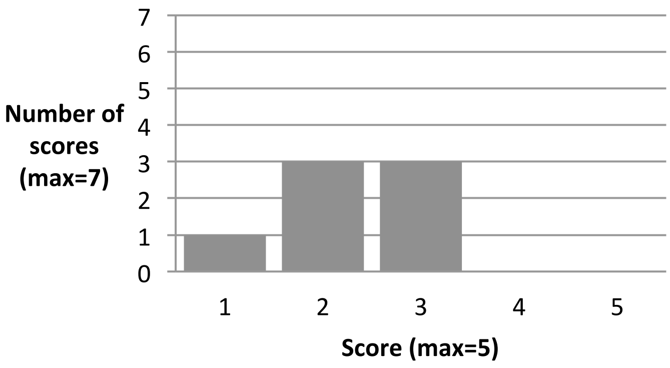

Comments from R1 Rankings: More evaluation needed • Only relevant for SCI •

**Measure ID**	**3**
**Name**	**Hierarchical Balance Short Forms (HBSF)**
**Reference**	Hou et al. Arch Phys Med Rehabil. 2011
**Purpose**	To assess balance function precisely in people with stroke with limited assessment burden
**# of Items**	16
**Evaluation Parameters**	Continuous (binary counts transformed into continuous measure)
**# of Categories**	N/A
**Graded Progression**	Yes, within each of three categories

**Mean Round 1 Results**	
**Psychometric**	1.9
**Feasibility**	3.4
**Overall**	2.4

Psychometric Score Distribution

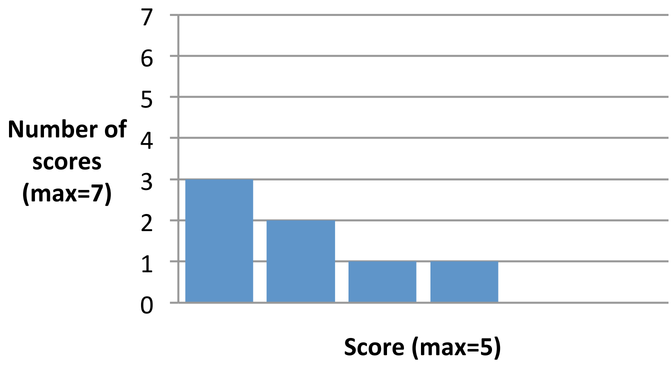

Feasibility Score Distribution

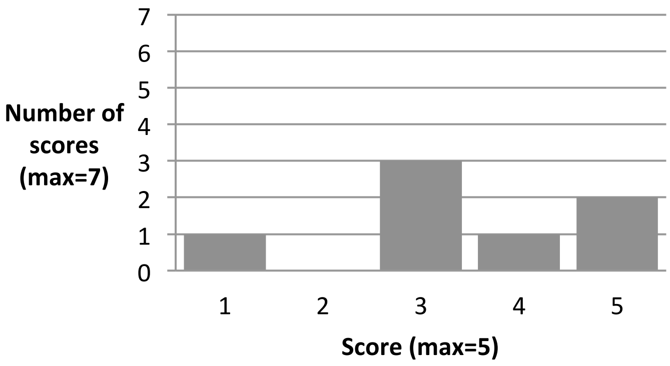

Comments from R1 Rankings: Very limited information, nothing on reliability etc and stroke specific, likely to be cheap but nothing else on time to administer • Staff may need special training to separate patients into three groups for testing • Large development sample (n=764) plus moderate sample for psychometric assessment (n=85). Contains mostly simple, non-challenging items •

Round 1 Results: Exclude (n=15)

**Measure ID**	**5**
**Name**	**Modified Balance Error Scoring System (M-BESS)**
**Reference**	Hunt et al. Clin Journal Sport Med. 2009
**Purpose**	To easily administer an objective assessment tool in a cost effective way
**# of Items**	4
**Evaluation Parameters**	Continuous (number of errors)
**# of Categories**	N/A
**Graded Progression**	No

**Mean Round 1 Results**	
**Psychometric**	1.3
**Feasibility**	3.4
**Overall**	1.5

Psychometric Score Distribution

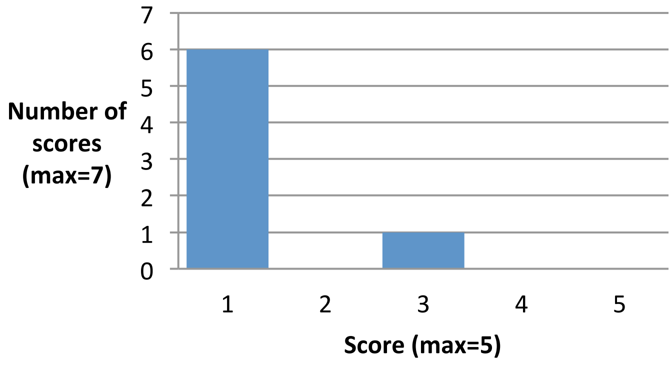

Feasibility Score Distribution

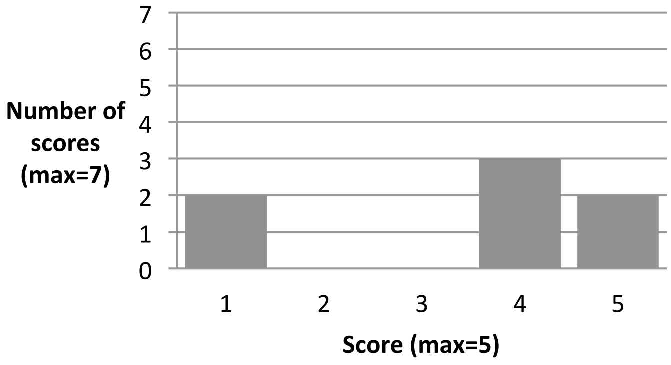

Comments from R1 Rankings: Why not MCTSIB? EO & EC • Administered to young athletes. Foam tests extend range of difficulty •

**Measure ID**	**15**
**Name**	**Clinical Gait and Balance Scale (GABS)**
**Reference**	Thomas et al. J Neurol Sci. 2004
**Purpose**	To comprehensively measure all essential elements of gait and balance
**# of Items**	18
**Evaluation Parameters**	Categorical
**# of Categories**	10 items have 5 levels, 4 items have 3 levels, 2 items have 2 levels, 2 items have subgroups with multiple categories
**Graded Progression**	No

**Mean Round 1 Results**	
**Psychometric**	1.7
**Feasibility**	2.8
**Overall**	1.5

Psychometric Score Distribution

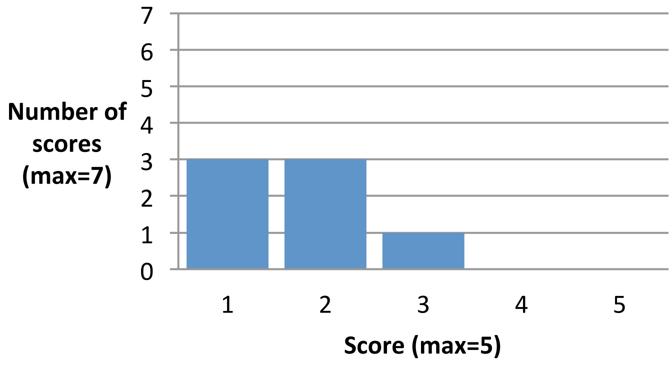

Feasibility Score Distribution

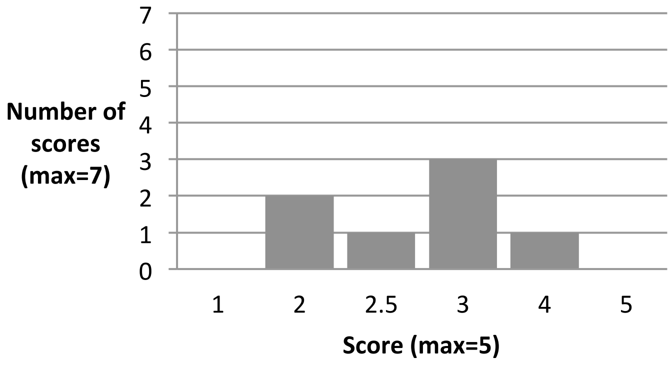

Comments from R1 Rankings: PD only (incl freezing) and limited reliability • Too many items • Cumbersome test, developed in small sample with PD •

Round 1 Results: Exclude (n=15)

**Measure ID**	**29**
**Name**	**Modified Figure of Eight Test**
**Reference**	Jarnlo and Nordell. Phys Theor Pract. 2003
**Purpose**	To measure the ability to walk slightly in lateral direction to both sides in an eight in combination with a narrow step width
**# of Items**	1
**Evaluation Parameters**	Continuous (time and number of "oversteps")
**# of Categories**	N/A
**Graded Progression**	No

**Mean Round 1 Results**	
**Psychometric**	1.7
**Feasibility**	3.4
**Overall**	1.8

Psychometric Score Distribution

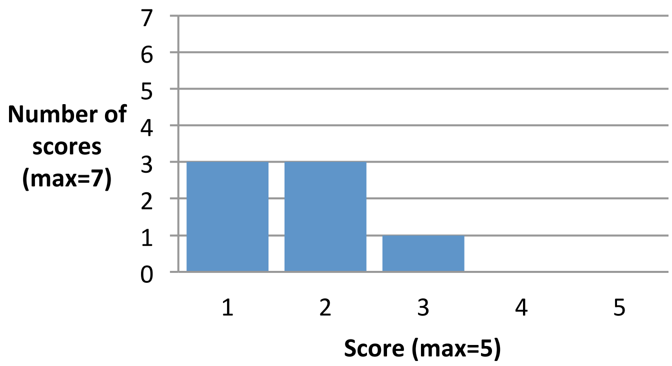

Feasibility Score Distribution

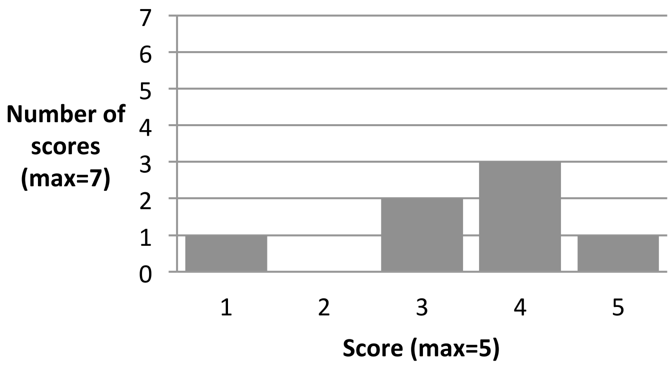

Comments from R1 Rankings: Inadequate evaluation: limited use for developing treatment plan • Nice simple performance test, which has some evidence of validity, but insufficient data on psychometric properties • One component •

**Measure ID**	**30**
**Name**	**Parallel Walk Test (PWT)**
**Reference**	Johansson et al. Physi Theor Pract. 1991
**Purpose**	To measure dynamic balance during gait
**# of Items**	3
**Evaluation Parameters**	Continuous [time and "footfall score" (+1 when part of foot placed on line, +2 when foot falls outside line or reached for something to maintain balance)]
**# of Categories**	N/A
**Graded Progression**	No

**Mean Round 1 Results**	
**Psychometric**	1.6
**Feasibility**	3.1
**Overall**	1.5

Psychometric Score Distribution

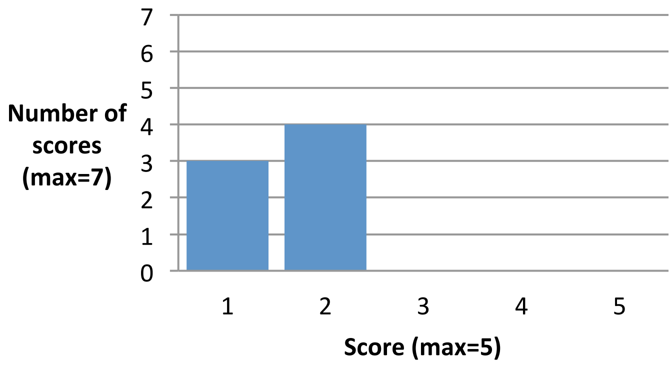

Feasibility Score Distribution

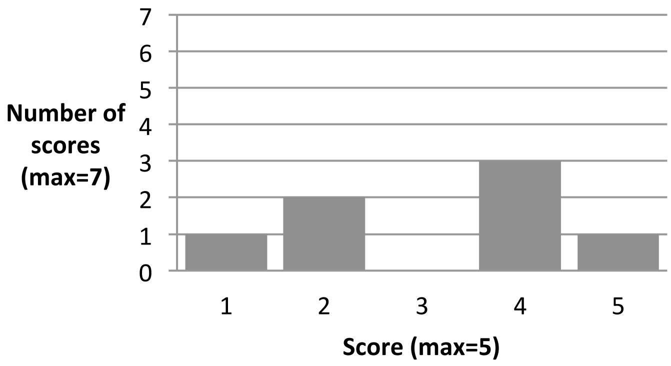

Comments from R1 Rankings: May be useful as screening tool although insufficient evaluation at this time • only in frail fallers, big range in ICCs so not sure on test-retest and will not easily show change • Insufficient data on psychometric properties • One component •

**Measure ID**	**32**
**Name**	**Modified Performance Oriented Mobility Assessment**
**Reference**	Fox et al. Arch Phys Med Rehabil. 1996
**Purpose**	To characterize recovery in physical capacity and functional independence after hip fracture
**# of Items**	13
**Evaluation Parameters**	Continuous (time, angle, distance, contact between thigh and abdomen)
**# of Categories**	N/A
**Graded Progression**	Yes for some tasks

**Mean Round 1 Results**	
**Psychometric**	1.5
**Feasibility**	2.7
**Overall**	1.8

Psychometric Score Distribution

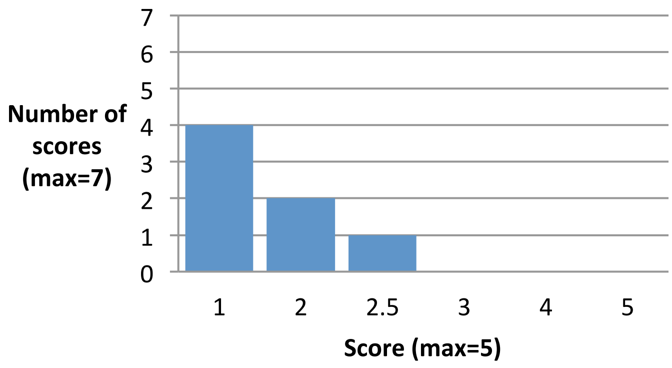

Feasibility Score Distribution

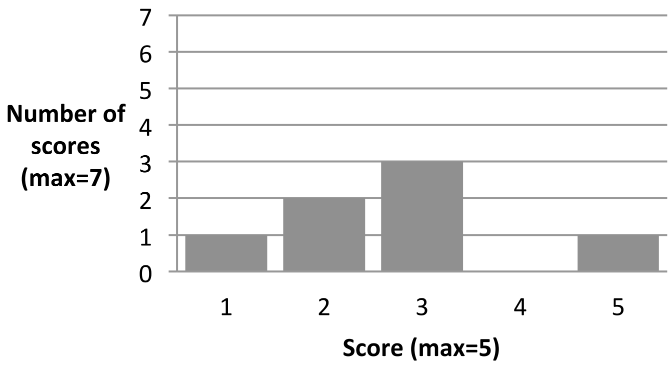

Comments from R1 Rankings: Good tool for use by trained professionals for post hip fracture patients • Insufficient data on psychometric properties • Small sample (n=23) post hip fracture •

**Measure ID**	**37**
**Name**	**Rapid Step Test (RST)**
**Reference**	Medell et al. J Geron A Biol Sci Med Sci. 2000
**Purpose**	To assess maximal and rapid stepping for balance and fall risk
**# of Items**	8
**Evaluation Parameters**	Continuous (step length, distance and time)
**# of Categories**	N/A
**Graded Progression**	No

**Mean Round 1 Results**	
**Psychometric**	1.7
**Feasibility**	3.0
**Overall**	2.3

Psychometric Score Distribution

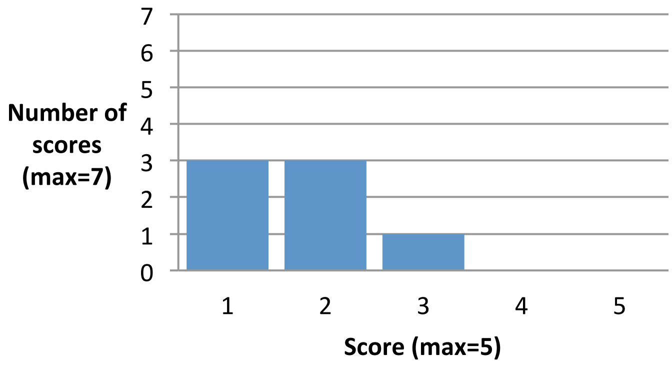

Feasibility Score Distribution

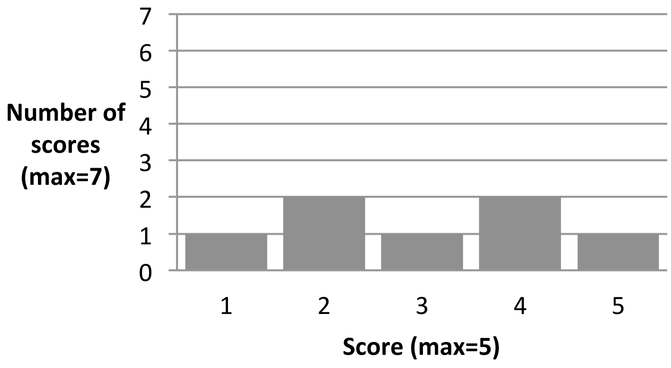

Comments from R1 Rankings: Insufficient evaluation of tool • Inadequate evidence on psychometric properties • Small study total (N=34) • See some value in this task more generally •

**Measure ID**	**39**
**Name**	**Head-Shake Sensory Organization Test (HS-SOT)**
**Reference**	Pang et al. Phys Ther. 2011
**Purpose**	To enhance the SOT to improve delineation of balance performance
**# of Items**	6
**Evaluation Parameters**	Continuous (equilibrium score as percentage from 0 - 100%)
**# of Categories**	N/A
**Graded Progression**	No

**Mean Round 1 Results**	
**Psychometric**	2.0
**Feasibility**	1.9
**Overall**	1.4

Psychometric Score Distribution

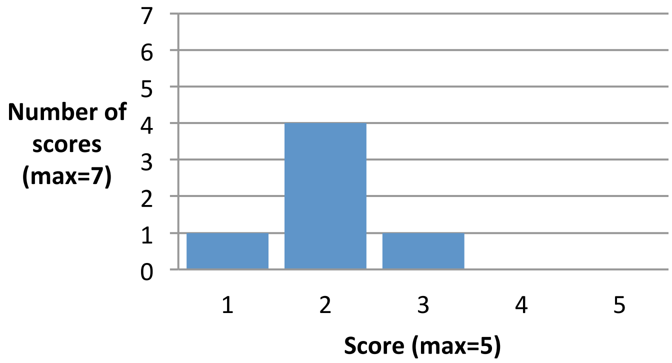

Feasibility Score Distribution

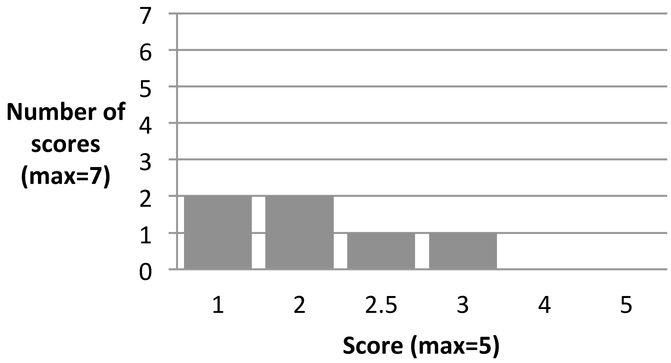

Comments from R1 Rankings: Requires expensive force equipment, limited validity, no idea how long to do • Short but of limited value • Not for all patients, poor theory •

**Measure ID**	**42**
**Name**	**Single Leg Hop Stabilization Test**
**Reference**	Riemann et al. J Sport Rehabil. 1999
**Purpose**	To assess postural control during a functional performance task
**# of Items**	20
**Evaluation Parameters**	Categorical
**# of Categories**	2
**Graded Progression**	Yes

**Mean Round 1 Results**	
**Psychometric**	1.9
**Feasibility**	2.7
**Overall**	2.0

Psychometric Score Distribution

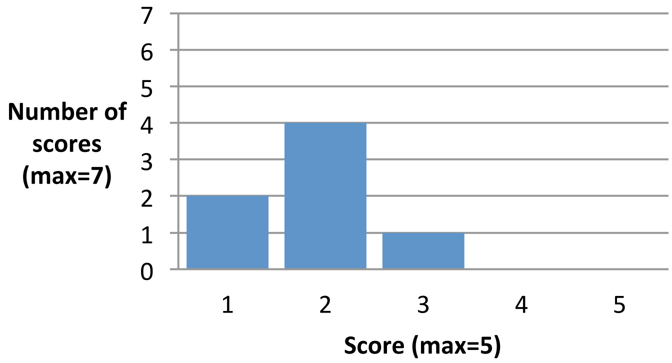

Feasibility Score Distribution

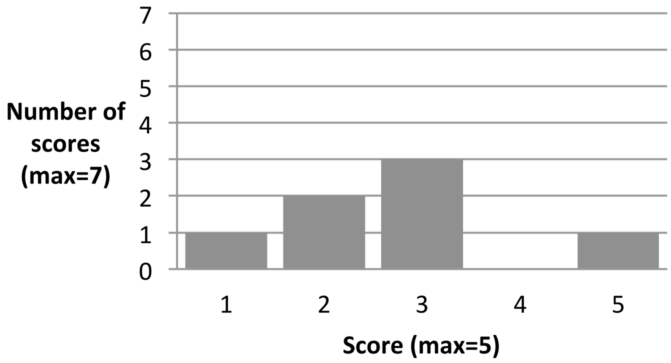

Comments from R1 Rankings: Insufficient evaluation of tool • Most OP cannot hop!, reliability tested on young people • Inadequate evidence for psychometric qualities • Impractical and dangerous •

**Measure ID**	**45**
**Name**	**Standing Test for Imbalance and Disequilibrium (SIDE)**
**Reference**	Teranishi et al. Jap J Comp Rehabil Sci. 2010
**Purpose**	To classify static standing balance ability for fall prevention
**# of Items**	4
**Evaluation Parameters**	Categorical
**# of Categories**	task 1: 2, task 2: 2, task 3: 3, task 4: 2
**Graded Progression**	Yes

Mean Round 1 Results

**Psychometric**	1.8
**Feasibility**	3.5
**Overall**	1.8

Psychometric Score Distribution

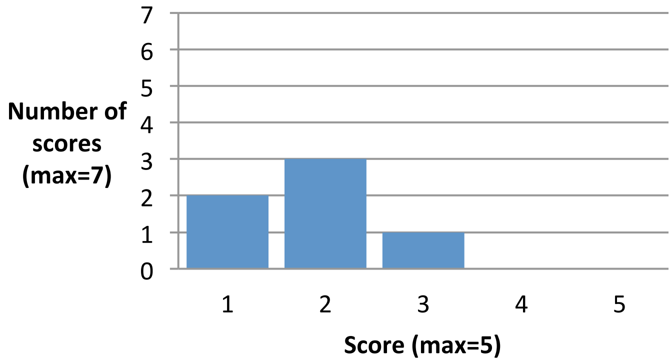

Feasibility Score Distribution

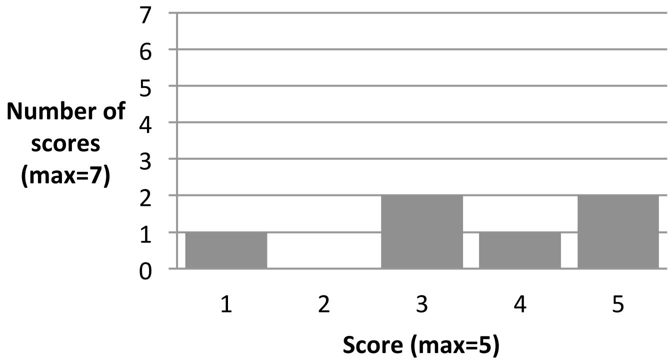

Comments from R1 Rankings: Insufficient evaluation of tool • See some value in this approach to assessment of static balance •

**Measure ID**	**46**
**Name**	**Star Excursion Balance Test (SEBT)**
**Reference**	Hertel et al. J Sport Rehabil. 2000
**Purpose**	To challenge the postural control systems of well-conditioned, physically active individuals recovering from lower extremity injuries
**# of Items**	8
**Evaluation Parameters**	Continuous (distance)
**# of Categories**	N/A
**Graded Progression**	No

Mean Round 1 Results

**Psychometric**	1.5
**Feasibility**	2.8
**Overall**	1.4

Psychometric Score Distribution

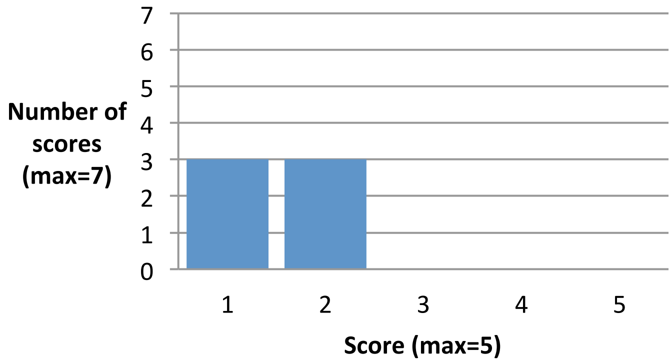

Feasibility Score Distribution

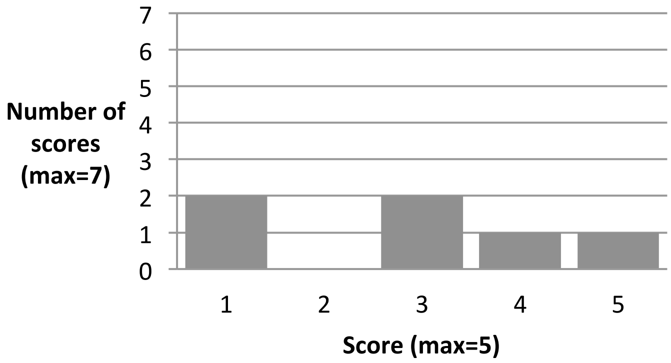

Comments from R1 Rankings: Insufficient evaluation of tool • Only suitable for young, very hard physical test, not brilliant ICCs for young people ;) •

**Measure ID**	**53**
**Name**	**Timed Up-and-Go Assessment of Biomechanical Strategies (TUG-ABS)**
**Reference**	Faria et al. J Rehabil Med. 2013
**Purpose**	To systematically evaluate biomechanical strategies used during performance of the TUG test
**# of Items**	15
**Evaluation Parameters**	Categorical
**# of Categories**	3
**Graded Progression**	No

Mean Round 1 Results

**Psychometric**	1.6
**Feasibility**	2.6
**Overall**	1.5

Psychometric Score Distribution

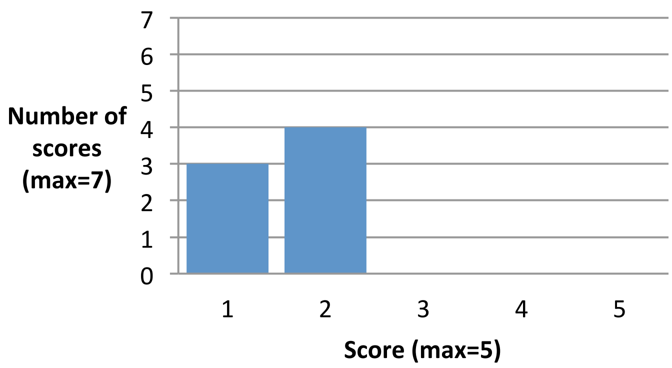

Feasibility Score Distribution

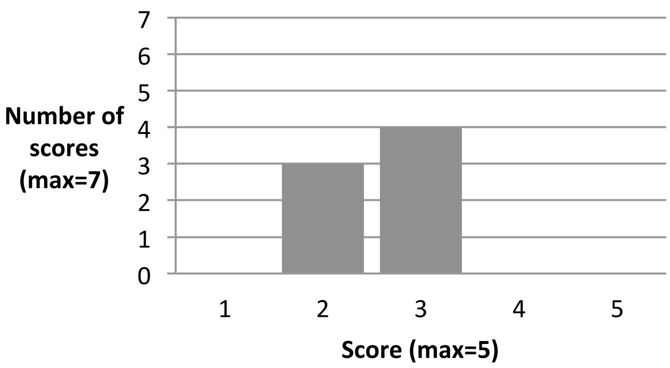

Comments from R1 Rankings: Cumbersome tool that needs much more evaluation •

**Measure ID**	**54**
**Name**	**Posture and Posture Ability Scale (PPAS)**
**Reference**	Rodby-Bousquet et al. Clin Rehab. 2012
**Purpose**	To evaluate posture and postural ability in people with severe disabilities
**# of Items**	4 tasks, 53 items assessed
**Evaluation Parameters**	Categorical scale
**# of Categories**	7 categories for postural ability, 2 categories for quality of posture
**Graded Progression**	No

Mean Round 1 Results

**Psychometric**	1.9
**Feasibility**	3.1
**Overall**	1.8

Psychometric Score Distribution

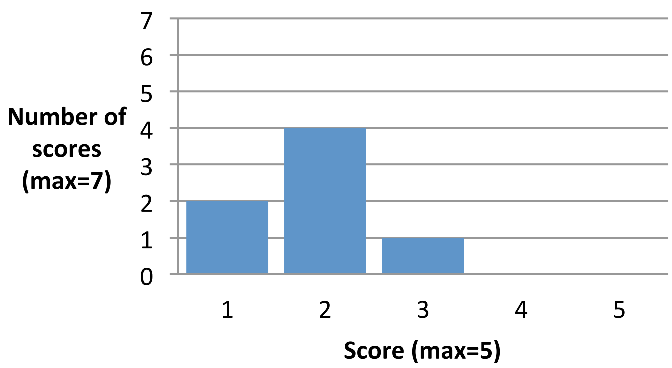

Feasibility Score Distribution

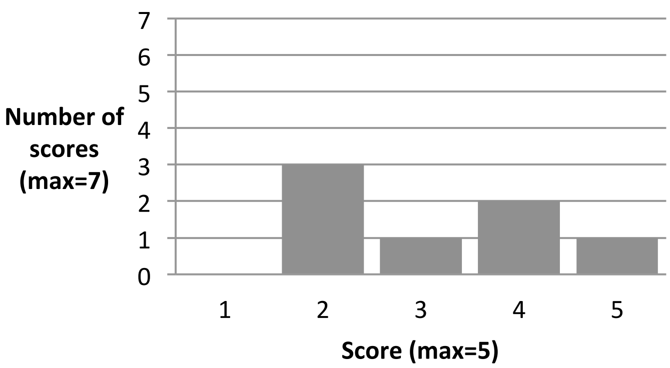

Comments from R1 Rankings: Limited use: CP; lacks psychometric rigor • CP patients, obvious ceiling effect •

**Measure ID**	**56**
**Name**	**Cross Step Moving on Four Spots Test (CSFT)**
**Reference**	Yamaji & Demura Arch Phys Med Rehabil 2013
**Purpose**	To evaluate crossover steps in older adults
**# of Items**	9
**Evaluation Parameters**	Continous (time to complete 9 steps)
**# of Categories**	N/A
**Graded Progression**	No

**Mean Round 1 Results**	
**Psychometric**	2.0
**Feasibility**	2.1
**Overall**	1.3

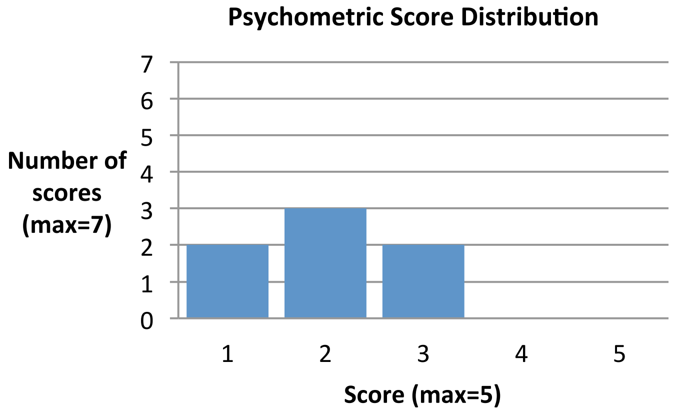

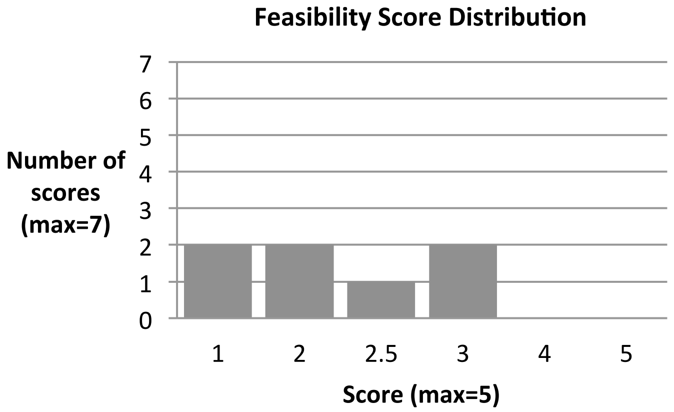

Comments from R1 Rankings: Requires a computer. May put those with poor balance at risk for falls during rapid stepping in changing directions • Limited •
