# Supplementary material for: Recommendations for a Core Outcome Set for Measuring Standing Balance in Adult Populations: A Consensus-Based Approach
Source: PLoS One. 2015 Mar 13;10(3):e0120568. doi: 10.1371/journal.pone.0120568 (PMC4358983; doi:10.1371/journal.pone.0120568)
Supplement: S2 File — This file contains the results of the second round of voting, including the criterion calculations for moving to round three. (PDF) [file pone.0120568.s002.pdf]

Round 2 Voting Results

Rate, on a scale of 1 (lowest) to 5 (highest), the overall suitability of each measure for inclusion in a balance Core Outcome Set for adult populations.	Score (N) 1	2	3	4	5	Rating Average	Number of ratings >=4	Decision (forward to round 3 if >=7 votes >=4)
ID #4: Balance Error Scoring System (BESS)	11	3	0	0	0	1.21	0	Exclude
ID #6: Balance Evaluation Systems Test (BESTest)	0	3	7	4	0	3.07	4	Exclude
ID #7: Brief Balance Evaluation Systems Test (Brief BESTest)	1	6	3	4	0	2.71	4	Exclude
ID #8: Mini Balance Evaluation Systems Test (Mini BESTest)	0	2	2	6	4	3.86	10	Retain
ID #12: Short Form of the Berg Balance Scale (SFBBS)	4	2	5	3	0	2.50	3	Exclude
ID #17: Community Balance and Mobility Scale (CB&M)	7	5	1	1	0	1.71	1	Exclude
ID #19: Dynamic Gait Index (DGI)	0	5	6	2	1	2.93	3	Exclude
ID #21: Functional Gait Assessment (FGA)	1	8	3	2	0	2.43	2	Exclude
ID #22: Five Times Sit-to-Stand Test (5STS)	4	6	0	4	0	2.29	4	Exclude
ID #23: Four Square Step Test (FSST)	2	3	6	2	1	2.79	3	Exclude
ID #24: Fullerton Advanced Balance (FAB) Scale	0	2	7	5	0	3.21	5	Exclude
ID #25: Functional Reach Test (FRT)	5	8	0	1	0	1.79	1	Exclude
ID #26: Multidirectional Reach Test	4	7	3	0	0	1.93	0	Exclude
ID #31: Performance Oriented Mobility Assessment (POMA)	0	5	4	4	1	3.07	5	Exclude
ID #40: Short Physical Performance Battery (SPPB)	0	1	4	8	1	3.64	9	Retain
ID #50: Expanded Timed Up-and-Go Test	9	5	0	0	0	1.36	0	Exclude
ID #52: Unified Balance Scale (UBS)	0	3	4	7	0	3.29	7	Retain
ID #55: High Level Mobility Assessment Tool (HiMAT)	11	2	1	0	0	1.29	0	Exclude
