# Supplementary material for: Recommendations for a Core Outcome Set for Measuring Standing Balance in Adult Populations: A Consensus-Based Approach
Source: PLoS One. 2015 Mar 13;10(3):e0120568. doi: 10.1371/journal.pone.0120568 (PMC4358983; doi:10.1371/journal.pone.0120568)
Supplement: S3 File — This file contains the number of votes received for each measure and the decision. (PDF) [file pone.0120568.s003.pdf]

Round Three Results

This measure should be included in the balance COS for adult populations.	Yes	No	Decision (include if >=9 votes =yes)
ID #11: Berg Balance Scale (BBS)	11	1	Include
ID #49: Timed Up-and-Go (TUG)	3	9	Exclude
ID #8: Mini Balance Evaluation Systems Test (Mini BESTest)	11	1	Include
ID #40: Short Physical Performance Battery (SPPB)	6	6	Exclude
ID #52: Unified Balance Scale (UBS)	0	12	Exclude
